# Jarid2 Methylation via the PRC2 Complex Regulates H3K27me3 Deposition during Cell Differentiation

**DOI:** 10.1016/j.molcel.2014.12.020

**Published:** 2015-03-05

**Authors:** Serena Sanulli, Neil Justin, Aurélie Teissandier, Katia Ancelin, Manuela Portoso, Matthieu Caron, Audrey Michaud, Berangère Lombard, Simao T. da Rocha, John Offer, Damarys Loew, Nicolas Servant, Michel Wassef, Fabienne Burlina, Steve J. Gamblin, Edith Heard, Raphaël Margueron

**Affiliations:** 1 Institut Curie, 26 Rue d’Ulm, 75005 Paris, France; 2 INSERM U934, 26 Rue d’Ulm, 75005 Paris, France; 3 CNRS UMR3215, 26 Rue d’Ulm, 75005 Paris, France; 4 MRC National Institute for Medical Research, The Ridgeway, London, Mill Hill NW7 1AA, UK; 5 INSERM U900, 26 Rue d’Ulm, 75005 Paris, France; 6 Sorbonnes Universités, UPMC Univ Paris 06, CNRS, ENS, UMR7203 LBM, 4 Place Jussieu, 75005 Paris, France; 7 Laboratory of Proteomics and Mass Spectrometry, 26 Rue d’Ulm, 75005 Paris, France; 8 Mines ParisTech, 35 Rue Saint Honoré, 77305 Fontainebleau, France

## Abstract

Polycomb Group (PcG) proteins maintain transcriptional repression throughout development, mostly by regulating chromatin structure. Polycomb Repressive Complex 2 (PRC2), a component of the Polycomb machinery, is responsible for the methylation of histone H3 lysine 27 (H3K27me2/3). Jarid2 was previously identified as a cofactor of PRC2, regulating PRC2 targeting to chromatin and its enzymatic activity. Deletion of Jarid2 leads to impaired orchestration of gene expression during cell lineage commitment. Here, we reveal an unexpected crosstalk between Jarid2 and PRC2, with Jarid2 being methylated by PRC2. This modification is recognized by the Eed core component of PRC2 and triggers an allosteric activation of PRC2’s enzymatic activity. We show that Jarid2 methylation is important to promote PRC2 activity at a locus devoid of H3K27me3 and for the correct deposition of this mark during cell differentiation. Our results uncover a regulation loop where Jarid2 methylation fine-tunes PRC2 activity depending on the chromatin context.

## Highlights

• PRC2 methylates Jarid2 on K116
• Jarid2 methylation promotes PRC2 activity
• H3K27me3 and Jarid2-K116me3 bind to the aromatic cage of Eed
• Jarid2 methylation regulates H3K27me3 deposition during ESC differentiation

## Graphical Abstract

**Figure:**
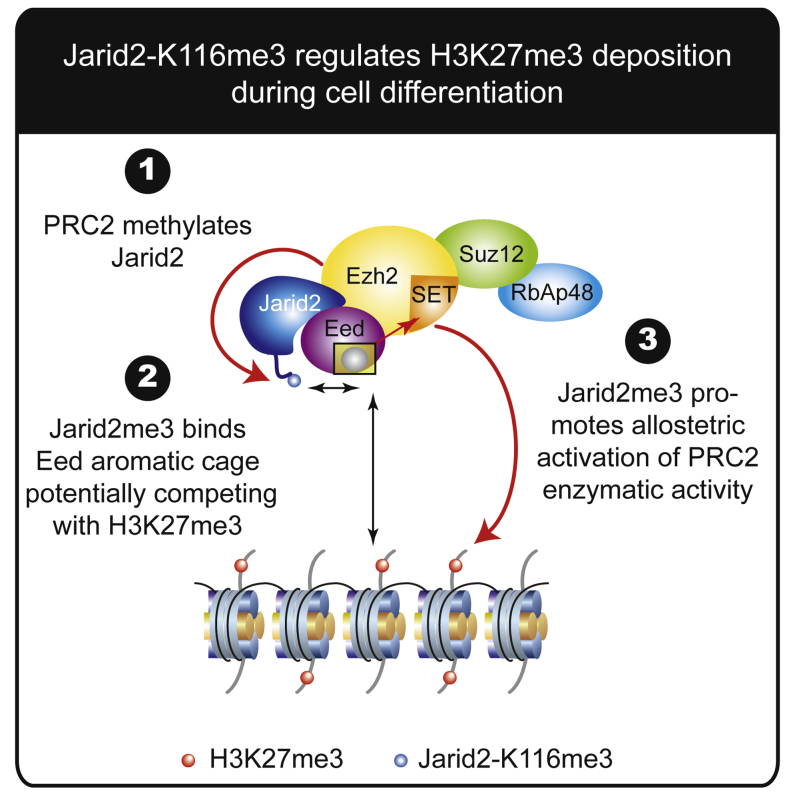

## Teaser

Methylation of H3K27 by the Polycomb complex PRC2 is essential for maintaining transcriptional silencing. Sanulli et al. report that Jarid2, a cofactor of this complex is also methylated by PRC2. Reciprocally, methylated Jarid2 regulates PRC2 enzymatic activity creating a regulatory loop.

## Introduction

Appropriate gene expression patterns in distinct cell lineages need to be set during embryogenesis and perpetuated during the lifespan of an organism. Polycomb group (PcG) proteins are known to take part in the maintenance of gene repression mostly through chromatin regulation (Margueron and Reinberg, 2011). Polycomb Repressive Complex 2 (PRC2), a key component of the Polycomb machinery, is composed of four core components: the catalytic subunit Ezh1/2, Suz12, Eed, and RbAp46/48. PRC2 is responsible for the di- and tri-methylation of histone H3 at lysine 27 (H3K27me2/3), a histone mark that correlates with silent or poorly transcribed genomic regions (Simon and Kingston, 2013). In addition to the core components of PRC2, several cofactors were shown to interact with this complex and to modulate both its binding to chromatin and its enzymatic activity (Margueron and Reinberg, 2011).

The molecular mechanisms responsible for PRC2 recruitment to chromatin are still unclear. Two models have been proposed (Klose et al., 2013; Voigt et al., 2013). A first “instructive” model proposes that PRC2 recruitment relies either on transcription factors (TFs) or on long non-coding RNAs (lncRNAs). Several studies support this hypothesis, with the examples of the lncRNAs Xist (Maenner et al., 2010; Zhao et al., 2008), HOTAIR (Rinn et al., 2007), or Kcnq1ot1 (Pandey et al., 2008; Redrup et al., 2009) and of TFs such as YY1 (Palacios et al., 2010; Woo et al., 2010, 2013) or Snail (Herranz et al., 2008). However, the nature and the relevance of the interactions between PRC2 and lncRNAs or TFs are not yet clear (Brockdorff, 2013).

A second “responsive” model relies on the observation that chromatin structure modulates PRC2 recruitment and functions. Several studies have shown that PRC2 activity is regulated by marks already present on chromatin. For example, the H3K4me3 and H3K36me3 marks associated to active transcription are reported to prevent the methylation of H3K27 by PRC2 when present on the same histone tail (Schmitges et al., 2011; Voigt et al., 2012; Yuan et al., 2011). In contrast, PRC2 enzymatic activity is stimulated by H3K27me3 via specific interactions between the methylated lysine 27 and the aromatic cage of Eed (Margueron et al., 2009) and by H2A ubiquitination (H2AUb) through a less defined molecular mechanism (Blackledge et al., 2014; Cooper et al., 2014; Kalb et al., 2014).

Other chromatin features have also been shown to impact PRC2/chromatin interaction such as DNA methylation (Bartke et al., 2010) and nucleosome density (Simon and Kingston, 2013; Yuan et al., 2012). In addition, PRC2 cofactors actively contribute to “sensing” chromatin structure. For instance, the PCL proteins were recently reported to recognize H3K36me3 (Brien et al., 2012; Cai et al., 2013; Musselman et al., 2012; Qin et al., 2013) and both the cofactors Aebp2 and Jarid2 have putative DNA binding domains (Kim et al., 2004, 2009). Of note, transcription can modulate PRC2 function not only through its impact on chromatin but also through PRC2 interaction with nascent RNA transcripts (Davidovich et al., 2013; Kaneko et al., 2013; Kanhere et al., 2010).

Jarid2, a member of the *Jumonji* family of proteins (Klose et al., 2006), is a developmental regulator, which is necessary for proper mouse development and embryonic stem cell (ESC) differentiation (Landeira and Fisher, 2011). However, unlike other members of the *Jumonji* family of proteins, Jarid2 has no histone demethylase activity. Previous studies showed that it interacts with PRC2 complex (Landeira et al., 2010; Li et al., 2010; Pasini et al., 2010; Peng et al., 2009; Shen et al., 2009). PRC2 and Jarid2 mostly co-localize at chromatin in ESC (Landeira et al., 2010; Li et al., 2010; Pasini et al., 2010; Peng et al., 2009; Shen et al., 2009) and Jarid2 depletion reduces PRC2 enrichment at chromatin, leading to the hypothesis that Jarid2 may act to recruit PRC2 (Pasini et al., 2010). In support of this, we recently demonstrated that Jarid2 has a nucleosome-binding domain that stabilizes PRC2 binding to chromatin (Son et al., 2013), interaction that could be modulated by lncRNAs (Kaneko et al., 2014). Notably, reduced occupancy of PRC2 at chromatin in the absence of Jarid2 does not translate into substantial decrease of H3K27me3 enrichment, suggesting that Jarid2 could constrain PRC2 enzymatic activity. However, several studies have now shown that Jarid2 positively regulates PRC2 activity (Li et al., 2010; Son et al., 2013; Zhang et al., 2011). Overall, although there is a consensus on the importance of Jarid2 as regulator of PRC2, how exactly it modulates PRC2 and H3K27me3 deposition is far from clear.

In this study, we investigate the interplay between Jarid2 and PRC2 and explore how this cofactor regulates H3K27me3 deposition and PRC2 function. We show that Jarid2 is a substrate for PRC2 and we characterize Jarid2 methylation in relation to PRC2 activity by a combination of biochemical, genomic, and in vivo approaches. Our study reveals that Jarid2 methylation participates in a unique regulatory mechanism controlling PRC2 catalytic activity and is required for the proper deposition of H3K27me3 during cell differentiation.

## Results

### PRC2 Methylates Jarid2 at K116 In Vitro

The function of PRC2 in maintaining gene silencing is thought to mostly rely on its methyltransferase activity toward H3K27. Hence, in vivo mutation of H3K27 to arginine leads to the same phenotypes as deletion of PRC2 components (Pengelly et al., 2013). By performing Lysine Methyl-Transferase (KMT) assays in vitro with PRC2 and Jarid2, we observed that Jarid2 is methylated by PRC2 both when this complex is reconstituted around Ezh2 or Ezh1 (Figure S1A).

**Figure 1:**
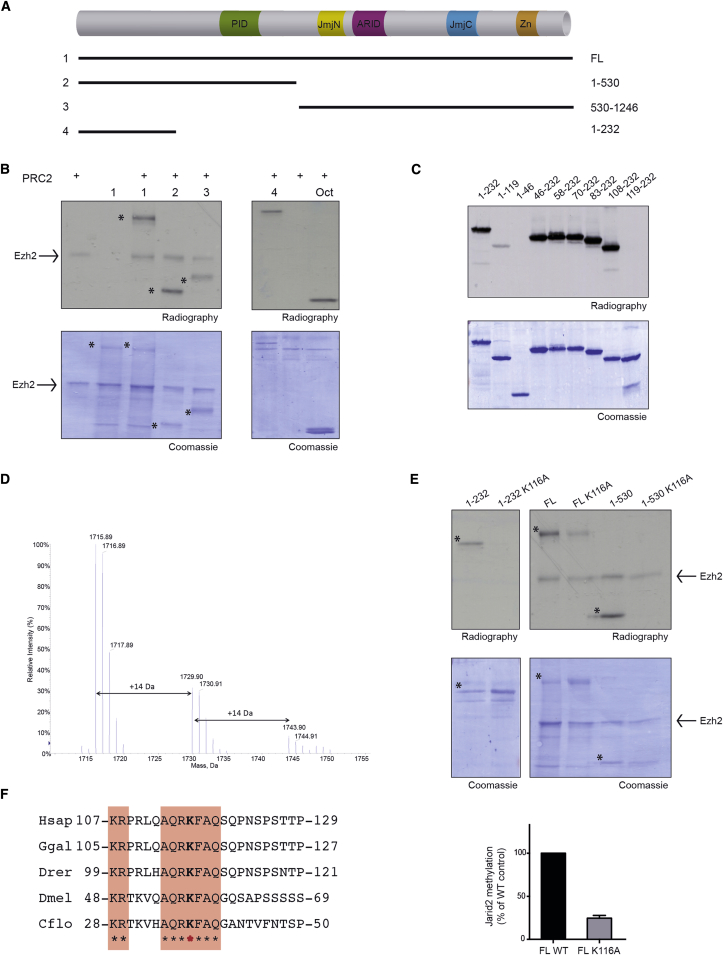
PRC2 Methylates Jarid2 at K116 In Vitro (A) Schematic representation of Jarid2 protein showing the annotated domains and the fragments used in KMT assays. PID, PRC2-interaction domain. (B) KMT assay performed with PRC2-Ezh2 as enzyme and either Jarid2 fragments described in (A) or octamers (Oct) as substrates. (C) KMT assay performed with PRC2-Ezh2 as enzyme and Jarid2 fragments as labeled on the top. (D) ESI mass spectrum showing non-(m/z 572.97), mono-(m/z 577.64), and di-(m/z 582.31) methylation at Lysine 116 in the peptide ^116^KFAQSQPNSPSTTPVK^131^ of Jarid2 fragment 1–232. (E) Top: KMT assay performed with PRC2-Ezh2 as enzyme and Jarid2 full-length (FL) and fragments WT or mutants K116A as substrates. (^∗^) indicates Jarid2 fragments. Bottom: quantification of FL Jarid2 methylation in percentage of Jarid2 WT (mean ± SD, n = 3). (F) Sequence conservation of the amino acids surrounding the methylated lysine across different species. See also Figure S1.

We produced different Jarid2 recombinant fragments (Figure 1A) that we used as substrates for in vitro KMT assays and we identified the main methylation site within Jarid2 1-232 N-terminal fragment (Figure 1B). Since the methylation is abrogated in the absence of amino acids (aa) 108 to 119 (Figure 1C) and thanks to mass spectrometry analysis, we identified that K116 is methylated in vitro (Figures 1D and S1B). Under the assay conditions, PRC2 catalyzed the mono- and di-methylation of Jarid2. We confirmed that Jarid2-K116 is the main target of PRC2 by generating a point mutation of K116 to alanine (K116A) in Jarid2 fragments or in the full-length protein (Figure 1E). Of note, we could detect the existence of a second minor site of methylation within the C-terminal of Jarid2, which represents about 20% of Jarid2 methylation signal (Figure 1B, fragment 3, Figure 1E). Since we could not confirm its existence in vivo (data not shown), we focused hereafter exclusively on the role of Jarid2-K116 methylation.

Given the conservation of the Polycomb machinery throughout evolution, we asked to what extent the K116 residue is conserved between different species. Strikingly, all the organisms expressing a homolog of Jarid2 that we analyzed harbored an identical stretch of seven amino acids centered on the methylated lysine (Figure 1F). In addition, PRC2 reconstituted with components from *Drosophila* was able to methylate a fragment of dJarid2 (formerly dJmJ) containing this conserved aa stretch surrounding the K46 residue (Figures S1C and S1D).

Our results therefore demonstrate that an evolutionary conserved lysine localized within the N terminus of Jarid2 is methylated in vitro by PRC2.

### Jarid2 Di- and Tri-Methylation Occurs In Vivo

**Figure 2:**
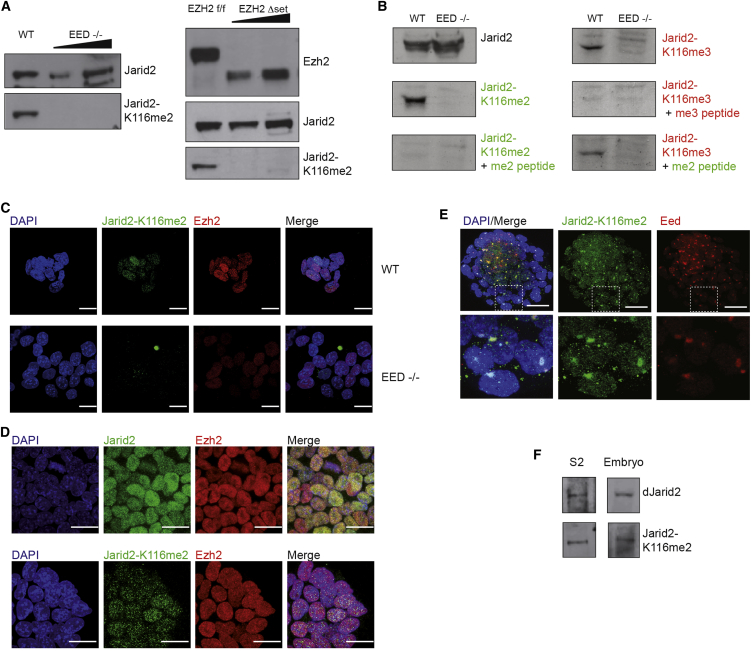
Jarid2 Methylation Occurs In Vivo (A) Left: western blot (WB) anti-Jarid2 and Jarid2-K116me2 on nuclear extract of E14 or Eed−/− ESC. Right: same in Ezh2 fl/fl-Rosa26-Cre-ERT2 before and after Tamoxifen treatment. Titrations correspond to 1× or 2× the amount of protein in controls. (B) WB and peptides competition experiment in ESC WT or Eed−/−. Nuclear extracts are probed with the indicated antibodies alone or in presence of the indicated peptides in 10-fold over antibody molar excess. (C) IF of Jarid2-K116me2 and Ezh2 on E14 and Eed−/− ESC. (D) IF of Jarid2 or Jarid-K116me2 and Ezh2 on E14 ESC. (E) IF in late mouse blastocyst for Eed and Jarid2-K116me2. Bottom panel is zoom-in of the top panel. Nuclei are stained with DAPI in all IF. (F) WB on whole extract of *Drosophila* S2 cell line and embryos probed with the indicated antibodies. See also Figure S2.

To test whether Jarid2 is methylated in vivo, we generated antibodies recognizing both Jarid2 di-methylated and tri-methylated at K116 (Jarid2-K116me2 and Jarid2-K116me3). We verified the specificity of these antibodies by dot blot on peptides and by western blot on in vitro methylated recombinant proteins (Figures S2A, S2B, and S2C). Western blot on nuclear extract of ESC line (E14) showed a robust signal, both for the di- and tri-methylated Jarid2-K116 antibodies, which was completely lost in ESC constitutively knocked out (KO) for *Eed* or conditionally KO for *Ezh2* (SET domain deletion) (Figure 2A). We additionally performed competition experiment with Jarid2-K116 me2 and me3 peptides and showed that the signal for Jarid2 tri-methylation was competed out by the Jarid2-K116me3 peptide but not by Jarid2-K116me2 peptide (Figure 2B).

After confirming that Jarid2-K116me2 signal is lost in *Eed−/−* ESC (Figure 2C), we compared the localization of the total and methylated form of Jarid2 by immunofluorescence (IF). We observed a broad nuclear staining for the total Jarid2, similar to the signal observed for Ezh2 (Figure 2D). In contrast, Jarid2-K116me2 shows a more punctuated signal, which overall does not overlap with DAPI-rich regions.

We have recently reported that Jarid2 is recruited during imprinted X chromosome inactivation in the pre-implantation female embryo and acts as bridge between PRC2 and Xist (da Rocha et al., 2014). We could also observe a robust signal for Jarid2-K116me2 by IF at the blastocyst stage, which was dramatically reduced in blastocysts expected to have undergone maternal loss of Ezh2 (Figure S2D). We then analyzed late female blastocyst and observed that Jarid2-K116me2 clearly co-localizes with the characteristic Eed cloud marking the inactive X chromosome (Figure 2E).

Consistent with our in vitro data suggesting that Jarid2 could also be methylated in other species, we could detect methylated dJarid2 by western blot on protein extracts from *Drosophila* S2 cells and embryos (Figure 2F). Moreover, polytene chromosome staining revealed that the methylated dJarid2 associates to chromatin and that it only partially co-localizes with the Polycomb protein Ph, in agreement with a previous report studying dJarid2 (Herz et al., 2012) (Figure S2E).

We conclude that Jarid2 is di- and tri-methylated by PRC2 at K116 in mammalian cells and that this post-translational modification occurs in vivo in particular during early mouse development. Finally, Jarid2 methylation is conserved through evolution as it also occurs in *Drosophila*.

### Jarid2-K116me2 Occupancy Co-Localizes with Total Jarid2 at Chromatin

**Figure 3:**
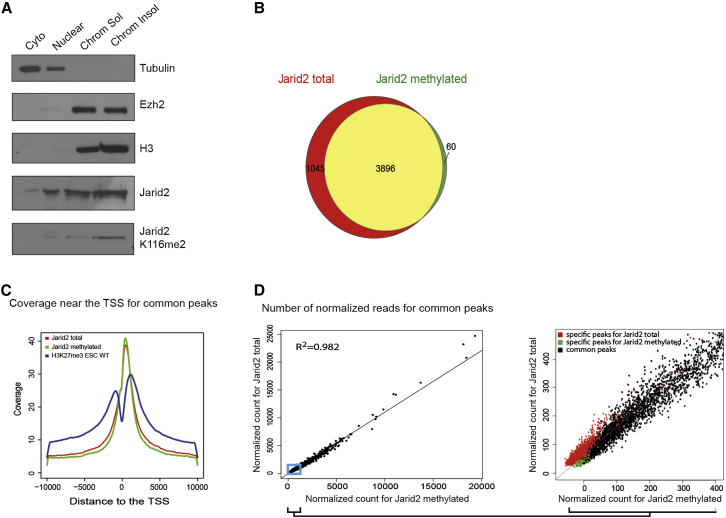
Jarid2 and Its Methylated Form Colocalize Almost Perfectly at Chromatin (A) Cellular fractionation of E14 ESC. Fractions are indicated on top and specific antibodies on the right. (B) Venn diagram showing the overlap (yellow) between peaks enriched for Jarid2 total and Jarid2-K116me2 or peaks specific for Jarid2 (red) or its methylated form (green) in E14 ESC. (C) Jarid2, Jarid2-K116me2, and H3K27me3 enrichments relative to TSS for common peaks. (D) Left: normalized read counts for Jarid2 versus Jarid2-K116me2 ChIP-seq at common target peaks. R^2^ = correlation coefficient. Right: zoom-in of the blue square area of left panel showing additionally read counts Jarid2 specific (red) and Jarid2-K116me2 specific (green). See also Figure S3.

The IF experiments prompted us to study in more details the distribution of Jarid2-K116me2 at chromatin. We first analyzed the subcellular distribution of Jarid2-K116me2 by performing cell fractionation experiments followed by western blots. Both total Jarid2 and Jarid2-K116me2 are preferentially enriched in the insoluble chromatin fraction, although they are also detected in the other nuclear fractions (Figure 3A).

We then performed ChIP-sequencing (ChIP-seq) experiments in E14 ESC to compare the localization of Jarid2 and of its methylated form. A similar number of reads were generated for both antibodies with more than 95% of reads mapping to the mouse genome (Figure S3A). As expected, almost all regions significantly enriched for Jarid2-K116me2 were also enriched for total Jarid2 (Figure 3B). Conversely, about 80% of the regions significantly enriched for total Jarid2 were enriched for its methylated form (Figure 3B). Jarid2 and Jarid2-K116me2 display the same narrow distribution around the transcription start sites (TSSs) of PRC2 target genes (Figure 3C), which contrast with the more widespread distribution of H3K27me3. Of note, the enrichments for Jarid2 and Jarid2-K116me2 correlate very well (Pearson correlation, R^2^ > 0,98, Figure 3D, left) and the peaks apparently specific either for total Jarid2 or for its methylated form are all characterized by a low number of reads for Jarid2 and H3K27me3 (Figures 3D, S3B, and S3C). Thus, rather than being specific to the methylated or total Jarid2, these specific peaks most likely result from the variability in sequencing depth and background estimation between the two samples.

In conclusion, the methylated form of Jarid2 localizes at chromatin of PRC2 target genes in ESC with a distribution that overlaps nearly perfectly with total Jarid2.

### Jarid2 Methylation Is Required for Efficient H3K27me3 Deposition

**Figure 4:**
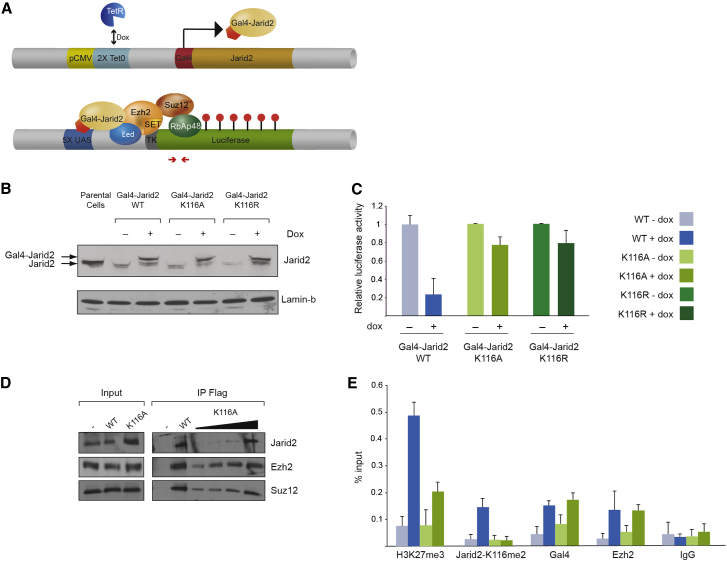
Jarid2 Methylation Is Required for Efficient H3K27me3 Deposition (A) Schematic representation of the inducible tethering system. The Luciferase reporter is downstream of a minimal TK promoter controlled by a 5× UAS sequence. Red arrows indicate the primers used for qPCR. (B) WB probing Jarid2 and lamin on nuclear extracts of T-rex 293 cells stably integrating the Gal4-Jarid2 (WT, K116A, and K116R) vectors and knocked down for Jarid2 through shRNA, prior and after 48 hr of doxycycline (dox) induction. (C) Relative luciferase activity in response to dox in the cell lines indicated on the x axis. Values represent the Relative Luciferase Activity normalized to the amount of protein (mean ± SD, n = 3). (D) Co-ImmunoPrecipitation (Co-IP) anti-Flag starting from nuclear extracts of HEK293 cell line control or overexpressing Flag-Jarid2 WT or K116A, followed by WB with the antibodies indicated on the right. (E) ChIP performed on T-rex 293 Gal4-Jarid2 WT and K116A cell lines. Antibodies used for ChIP are indicated on the x axis. y axis represents percent of input (mean ± SD, n ≥ 3). See also Figure S4.

It has been previously shown that artificial tethering of Jarid2 to a reporter gene induces transcriptional repression in a PRC2-dependent manner (Li et al., 2010; Pasini et al., 2010). We similarly engineered cell lines stably integrated with a luciferase reporter gene and expressing, in an inducible manner, Gal4-Jarid2 WT or mutants that cannot be methylated, in which K116 was replaced by an alanine or an arginine (K116A or K116R, Figure 4A). To avoid potential leakiness of the inducible system or contribution of the endogenous Jarid2, we knocked down both the endogenous and Gal4-fusion Jarid2 proteins by RNA interference. All cell lines present substantially reduced Jarid2 protein levels as shown by western blot (Figure 4B). Upon induction, while the Gal4-Jarid2 WT protein led to transcriptional repression of the luciferase reporter, both the Gal4-Jarid2-K116A and K116R only modestly reduced transcription (Figure 4C). To rule out the possibility that the K116 point mutation interfered with Jarid2 binding to PRC2, we performed co-immunoprecipitation in HEK293 cells overexpressing Flag-Jarid2 either WT or K116A mutant (Figure 4D). Both proteins pulled down PRC2 components with the same efficiency, indicating that PRC2-Jarid2 interaction is not affected by the K116A point mutation.

Monitoring H3K27me3 levels at the reporter transgene by ChIP, we found that the expression of Gal4-Jarid2 WT resulted in an increase of H3K27me3 by over 5-fold, while expression of Gal4-Jarid2-K116A/R only led to an increase of the mark by about 2-fold (Figures 4E and S4). Importantly, the Gal4 fusion proteins and Ezh2 were similarly recruited to the reporter upon doxycycline treatment (Figure 4E). Thus, mutating K116 of Jarid2 does not affect PRC2 recruitment per se but does affect its ability to promote PRC2 enzymatic activity on H3K27.

We conclude that Jarid2-K116 methylation is dispensable for PRC2 recruitment to chromatin but necessary to efficiently promote PRC2-mediated H3K27me3 deposition.

### Jarid2 Methylation Regulates PRC2 Enzymatic Activity Depending on the Chromatin Context

**Figure 5:**
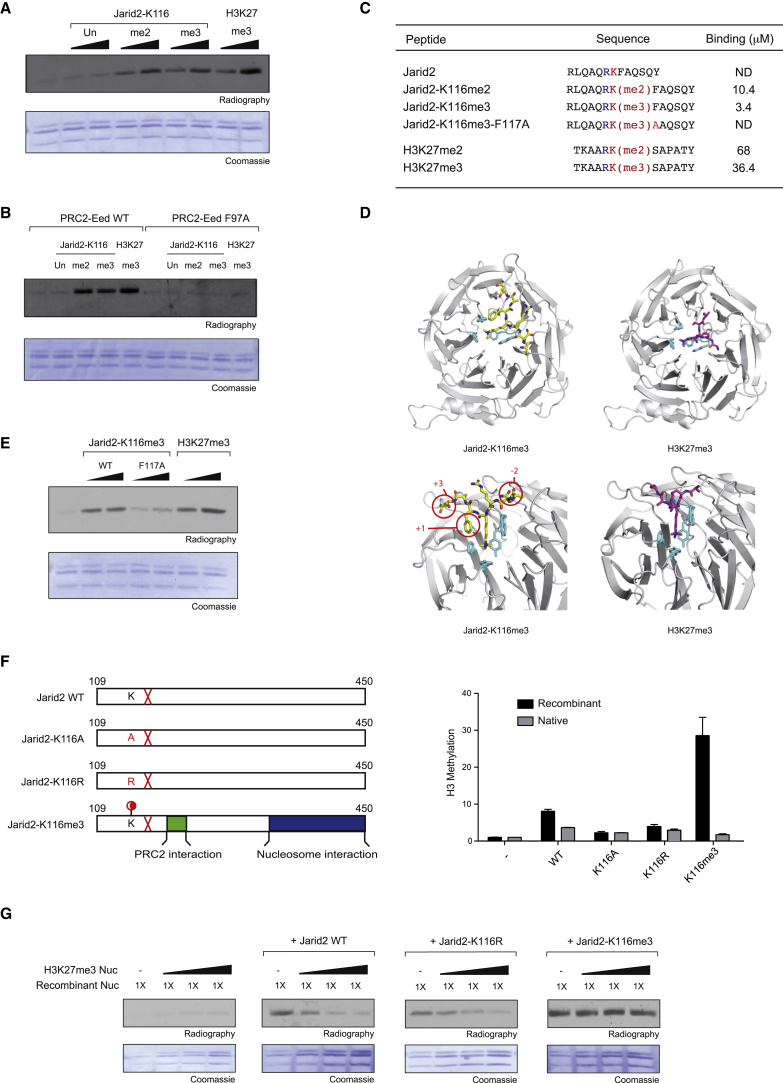
Jarid2 Methylation Regulates PRC2 Enzymatic Activity Depending on Chromatin Environment (A) HKMT assay performed with PRC2-Ezh2 on recombinant oligonucleosomes in the presence of peptides mimicking either Jarid2-K116me2 and me3 or H3K27me3. (B) HKMT assay as in (A) performed with PRC2-Ezh2 reconstituted with either Eed WT or F97A. (C) Table indicating the sequences of the peptides and the relative dissociation constant K_d_ (μM) for ΔEed binding measured by ITC. (D) Ribbons representation of ΔEed-Jarid2-K116me3 and ΔEed-H3K27me3 complexes. Eed is in gray; Jarid2 peptide is in yellow while H3 peptide is in purple, both in stick representation. Eed aromatic amino acids are in stick and cyan color. Top: top views. Bottom: zoom-in of side views. (E) HKMT assay as in (A) in presence of peptides Jarid2-K116me3 WT or F117A. (F) Left: representation of the chimeric proteins used in the HKMT assay. The red cross indicates the point mutations introduced in the protein to perform native chemical ligation. Red circle indicates K116me3. Right: quantification of PRC2-HKMT activity in the presence of the three Jarid2 fragments (mean ± SD, n = 3). Native (gray bars) or recombinant (black bars) chromatins were used as substrates. (G) HKMT assay performed with PRC2-Ezh2 in the absence or presence of Jarid2 WT, Jarid2-K116me3, or Jarid2 K116R. Recombinant chromatin reconstituted in presence of increasing amount of H3K27me3 octamers is used as substrate. See also Figure S5.

Our previous work showed that the C-terminal domain of Eed forms an aromatic cage that recognizes H3K27me3, leading to allosteric changes that stimulate PRC2 enzymatic activity on H3K27 (Margueron et al., 2009). To determine whether Jarid2 could be involved in a similar mechanism, we monitored PRC2 Histone-Lysine-Methyl Transferase (HKMT) activity in presence of peptides mimicking Jarid2-K116 di- and tri-methylation. We observed that both Jarid2-K116me2 and me3 peptides markedly stimulate PRC2 enzymatic activity on recombinant oligonucleosomes, similarly to the H3K27me3 peptide (Figure 5A). This stimulatory activity was lost with a recombinant PRC2 complex carrying a point mutation (Eed F97A) in Eed’s aromatic cage (Figure 5B), indicating specific interaction between the methylated K116 and the aromatic cage.

To confirm the specificity of Eed-Jarid2-K116me2/3 interaction, we measured the binding affinity of Eed (ΔEed residues 77 to 441, see [Margueron et al., 2009]) for Jarid2-K116me2/3 peptides using isothermal titration calorimetry (ITC). Jarid2-K116me3 peptide exhibited ten times more affinity for ΔEed than H3K27me3 peptide, with a dissociation constant (K_d_) of 3 μM (Figures 5C and S5). Jarid2-K116me2 peptide affinity for Eed was lower than Jarid2-K116me3 but still higher than H3K27me3.

To further explore Eed-K116me3 interactions, we solved the structure of ΔEed co-crystallized with Jarid2-K116me3 peptide and compared it with the structure of ΔEed-H3K27me3. Within the two structures, the Eed aromatic cages largely superimpose and the Jarid2-K116me3 and H3K27me3 peptide backbones significantly overlap, despite some differences in the aa sequences (Figure 5D; Table 1). However, Jarid2-K116me3 peptide forms additional contacts with Eed (Figure 5D, red circles). In particular, the large hydrophobic side chain of the phenylalanine at position +1 (F117) lies alongside the stem of the methyl-lysine and could contribute to the stabilization of K116me3 within the hydrophobic pocket. Indeed, a peptide in which the F117 was replaced by an alanine (F117A) did not interact with Eed (Figure 5C) and was unable to stimulate PRC2 enzymatic activity (Figure 5E).

The N-terminal region of Jarid2 comprises distinct domains for both PRC2 and chromatin interactions (Son et al., 2013). To evaluate the role of K116me3 in this context, we generated fragments of Jarid2 covering aa 109–450, K116 being fully methylated, unmodified, or mutated to alanine or arginine. The fully methylated Jarid2 fragment was produced by coupling, through native chemical ligation, a peptide containing aa 109–123 (K116me3) with the aa 124–450 fragment. We then monitored PRC2 enzymatic activity on recombinant or native nucleosomes either alone or in the presence of equimolar amounts of Jarid2 fragments (Figure 5F). As expected, all Jarid2 fragments stimulated PRC2 enzymatic activity. However, the methylated fragment was about 5- to 6-fold more efficient as compared to the K116A or K116R fragments when recombinant chromatin was used as substrate (Figure 5F, black bars). In contrast, this stimulation was lost when native chromatin was used as substrate (Figure 5F, gray bars). This result suggests that histones PTMs present on native chromatin could interfere with the binding of the methylated K116 to Eed’s aromatic cage.

To evaluate a potential competition between H3K27me3 and Jarid2-K116me3, we reconstituted chromatin in presence of increasing ratios of octamers fully methylated on K27 and monitored PRC2 activity in presence of Jarid2 fragments (Figure 5G). As expected, the titration of H3K27me3 stimulates PRC2 enzymatic activity. In presence of the methylated form of Jarid2, the titration of H3K27me3 has no effect on the stimulation of PRC2 mediated by Jarid2. In contrast, in presence of the non-methylated or non-methylatable forms of Jarid2, the titration of H3K27me3 limits Jarid2-mediated stimulatory effect. This result suggests the presence of a competition between the stimulatory activity of H3K27me3 and Jarid2. When methylated, Jarid2 controls PRC2 enzymatic activity even in presence of H3K27me3. In contrast, when Jarid2 is not methylated, H3K27me3 is taking over the control of PRC2 activity. Importantly, this assay addresses only the case of Jarid2 methylation versus trans-nucleosomes H3K27me3-mediated regulation of PRC2 enzymatic activity. The outcome of this competition could be different when additional histones PTMs or nucleosomes asymmetrically methylated on H3K27 are present.

Altogether, these experiments show that Jarid2 methylation is recognized by the aromatic cage of Eed and promotes allosteric activation of PRC2 enzymatic activity. Yet, this mechanism is modulated by the presence of PTMs on chromatin, most likely owing to the competitive binding to Eed.

### Jarid2 Methylation Is Required for PRC2 Function upon Cell Differentiation

**Figure 6:**
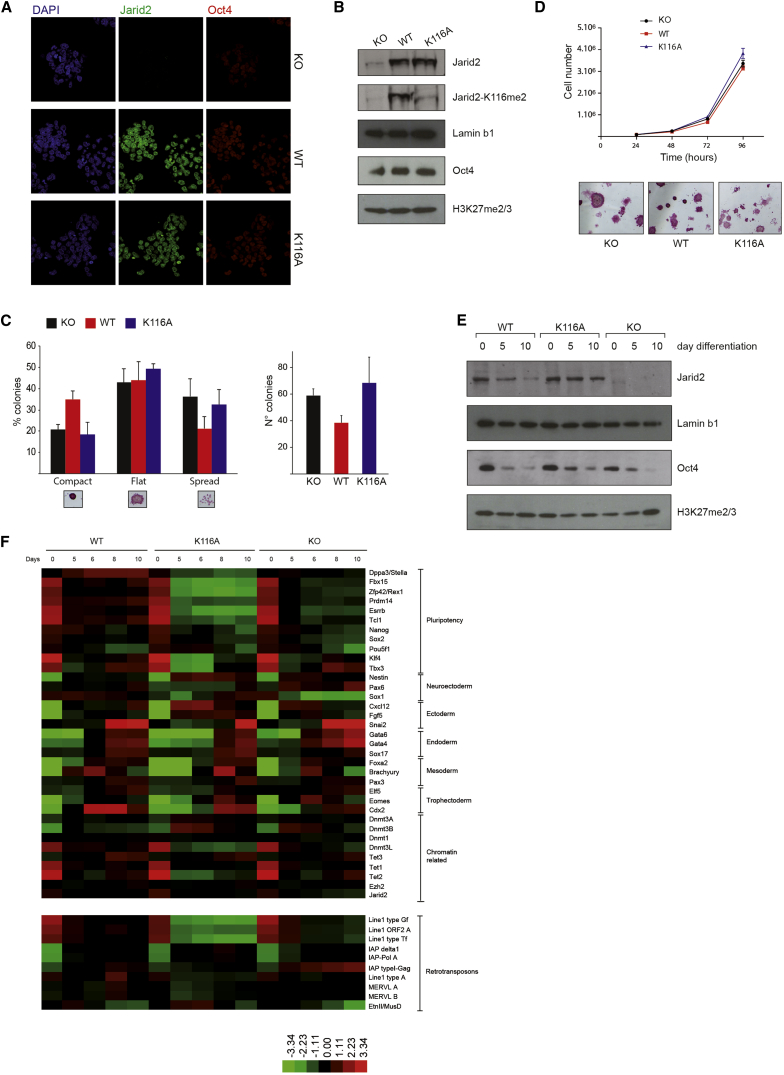
Jarid2 Methylation Is Required to Set Up Differentiation Programs in ESC (A) IF for Jarid2 and Oct4 in Jarid2 KO and Jarid2 WT or K116A rescued ESC. (B) WB with the indicated antibodies on nuclear extracts of the three ESC lines. (C) Left: colony morphology quantification of the indicated ESC. Values represent the percentage of alkaline-phosphatase-positive colonies showing a compact, flat, or spread morphology (mean ± SD, n ≥ 2). Right: number of methylene-blue-stained colonies (mean ± SD, n ≥ 2) detected 8 days after plating 100 cells per well. (D) Top: cell growth curve of the ESC over 4 days (mean ± SD, n = 3). Bottom: representative view of phosphatase alkaline staining. (E) WB probing nuclear extracts during ESC differentiation. (F) RNA transcript quantification through multiplexed-digital hybridization-based analysis (mean, n = 2). Heatmap representing the Log2-transformed median centered values. Top: messenger RNA classified by function. Bottom: transcripts from repeated sequences. Values are normalized around a set of housekeeping genes (ActB, Hprt1, Gapdh, Ppia, RplPO, and Rrm2). See also Figure S6.

The results presented so far demonstrate that Jarid2 methylation is important for PRC2 activity in vitro and when artificially tethered to chromatin in a cellular context. To confirm the biological relevance of this regulation, we evaluated the function of Jarid2 methylation in ESC. We used ESC KO for *Jarid2* (Shen et al., 2009) and generated cell lines rescued with either WT or K116A mutant forms of Jarid2. Since Jarid2 is dynamically regulated during cell differentiation, the rescues were performed using bacterial artificial chromosomes (BACs) in order to recapitulate the endogenous expression of the *Jarid2* gene. Rescued ESC lines expressed Jarid2 homogeneously, with appropriate nuclear distributions as illustrated by IF (Figure 6A) and at similar levels as shown by western blot (Figure 6B). Moreover, all three lines exhibited similar global levels of H3K27me3 and Oct4 protein (Figure 6B). Interestingly, Jarid2 WT rescued ESC presented slight morphological differences, displaying more typical ESC colonies, when compared to Jarid2 KO or K116A mutant cells that tend to show a more flattened morphology (Figure 6C, left). They also formed fewer colonies when plated at low density as compared to the K116A rescued line (Figure 6C, right). Nonetheless, all the three ESC lines showed similar levels of alkaline phosphatase activity, comparable proliferation rates, and no major differences in gene expression (Figure 6D and day 0 in Figure 6F), thus confirming that Jarid2 is not necessary for ESC pluripotency and self-renewal (Landeira et al., 2010; Shen et al., 2009).

Since Jarid2 depletion has been reported to cause pronounced defects during ESC differentiation (Landeira et al., 2010; Li et al., 2010; Shen et al., 2009), we induced differentiation of the three ESC lines into embryoid bodies (EBs). All ESC lines could initiate differentiation as illustrated by the decreased protein levels of Jarid2 and Oct4 at day 5 and 10 of differentiation (Figure 6E). Next, using digital multiplexed gene expression analysis (NanoString nCounter), we measured transcript levels, during the course of differentiation, of a set of markers of ESC pluripotency and differentiation toward the different germ layers. Overall, gene expression follows the same trend in all three cell lines but with marked differences in term of kinetics and/or extent of gene regulation (Figures 6F and S6). For instance, most pluripotency markers were downregulated (e.g., *Fbx15*, *Zfp42*, *Klf4*, *Essrb*, *Prdm14*, *Tcl1*); however, downregulation appears earlier and more pronounced in the KO and Jarid2-K116A lines, the latter displaying an even more marked effect. A noticeable exception is the transcript *Stella*/*dppa3*, which is upregulated in the Jarid2-WT line but downregulated in the two other lines upon differentiation.

KO and Jarid2-K116A lines also displayed specific differences. In particular, markers of differentiation toward the mesoderm such as *Brachyury* and *Foxa2* are activated later in the Jarid2 K116A cells. We confirmed this data by RT-qPCR and further observed that Jarid2 KO cells seem to more efficiently activate the expression of genes such as *Gata4* or *Olig1* (Figure S6). Finally, repeats were also found to be differentially regulated depending on Jarid2 status as illustrated by the LINE 1 elements (Figure 6F, bottom).

Taken together, these results indicate that Jarid2 and its methylation play an important role during the establishment of cellular differentiation programs.

### Jarid2 Is Required for Proper Localization of H3K27me3 during Cell Differentiation

**Figure 7:**
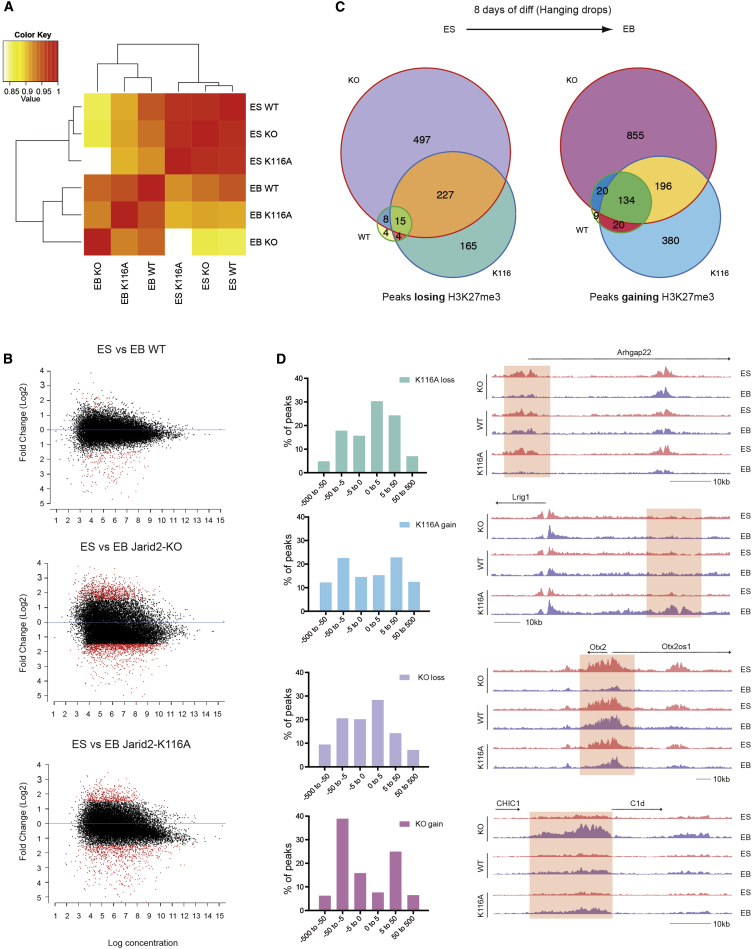
Jarid2 Is Required for the Proper Deposition of H3K27me3 during Cell Differentiation H3K27me3 ChIP-seq performed in ESC and EBs in the cell lines described in Figure 6. Each ChIP-seq was performed in biological duplicates. (A) Correlation heatmap based on all peaks found to be enriched for H3K27me3 in at least one of the six samples. (B) MA plot of ES-EB contrast, with peaks identified as significantly differentially bound shown in red. y axis shows Log2 fold change. x axis shows Log2 concentration reads. (C) Venn diagram representing peaks loosing or gaining H3K27me3 during differentiation. (D) Left: distance relative to TSS of the peak losing or gaining H3K27me3 specifically in the Jarid2 KO or K116A cells during differentiation. Right: genome browser images of representative genes from each of the categories described above, the same scale was used in all tracks. See also Figure S7 and Table S1.

To understand the misregulation of gene expression in the Jarid2 K116A and Jarid2 KO cells during differentiation, we evaluated the distribution of H3K27me3 by ChIP-seq in undifferentiated ESC (Jarid2 KO, WT-, and K116A- rescued) and in EBs at 8 days of differentiation. Sequencing was performed on two independent biological samples (Figure S7A). As shown in Table S1, good correlations between duplicate experiments are observed. We generated a hierarchical clustering based on the correlation of all peaks found enriched for H3K27me3 in at least one of the six samples (Figure 7A). ESC and EBs formed two very distinct clusters, thus confirming that all three lines engaged differentiation. H3K27me3 genomic distribution between all three ESC lines was strongly correlated and overall differences did not exceed the variability between duplicates (Table S1). This result is consistent with the lack of major effect on H3K27me3 upon *Jarid2* deletion in ESC and could explain some of the discrepancies among the reports describing the consequences of knocking down or out Jarid2 on H3K27me3 enrichment (Landeira et al., 2010; Li et al., 2010; Pasini et al., 2010; Peng et al., 2009; Shen et al., 2009). In contrast, we observed noticeable differences regarding the distribution of H3K27me3 in EBs depending on Jarid2 status. Overall, the H3K27me3 profiles diverged more when comparing H3K27me3 ChIP-seq between ES and EB in the lines knockout or mutant for Jarid2 (Figure 7B). Consequently, a substantial number of peaks are specifically gained or lost in the Jarid2 mutant lines as compare to the WT during differentiation (Figure 7C).

Genomic ontology (GO) analysis revealed that the peaks losing H3K27me3 display the canonical features of PRC2 target peaks such as strong enrichment for CpG-islands, 5′ UTRs, coding regions, or promoters (Figures 7D and S7B). In contrast, the H3K27me3 peaks that were gained in the Jarid2 KO and K116A cells are not characterized by such a clear enrichment for any specific category, except for a bias toward CpG-islands in the K116A cells. The distinct features of peaks that are gained and lost during transition from ES to EB in the Jarid2 KO and K116A lines is also highlighted by the analyses of the localization of peaks relative to the TSS (Figure 7D). While the majority of the peaks losing H3K27me3 in the mutant cells resides within 5 kb of a TSS, the peaks gaining H3K27me3 are more broadly distributed all over the genome. Hence, we found gains of H3K27me3 corresponding to a broad enrichment in between two genes as well as more localized peaks in genomic regions without any gene annotation (Figure 7D).

Altogether our ChIP-seq data highlight the crucial role of Jarid2 and its methylation for the proper deposition of H3K27me3 during the process of differentiation. We further show that the aberrant loss of H3K27me3 detected in the mutant cells appears to be compensated by gains in this mark elsewhere in the genome. Whereas the loss of H3K27me3-enriched sites in the Jarid2 mutant cells is likely to have transcriptional consequences considering the proximity of such sites to the TSS, it is much less clear what the impact of the peaks gaining H3K27me3 will have on gene expression considering their apparently more random distribution.

## Discussion

PRC2, as a key component of the PcG machinery, is essential to ensure cellular memory through the maintenance of transcriptional repression. This implies that its genomic targeting should be finely regulated and cell-type specific. However, the mechanisms regulating its activity remain largely obscure. Here, we report that Jarid2, which is known to directly interact with PRC2 and to co-localize genome-wide with this complex (Landeira et al., 2010; Li et al., 2010; Pasini et al., 2010; Shen et al., 2009), is actually a substrate of PRC2’s enzymatic activity. Furthermore, we have shown that Jarid2 methylation modulates the enzymatic activity of PRC2 creating a unique regulatory loop, which impacts on H3K27me3 localization. Consequently, a point mutant preventing Jarid2 methylation leads to defects in cell differentiation with inappropriate H3K27me3 deposition.

Until recently, it was assumed that the sole substrate of PRC2 was the histone H3K27. A few publications have now proposed that Ezh2 could methylate other non-histone substrates and in particular TFs such as GATA4, STAT3, the androgen receptor, and RORα (He et al., 2012; Kim et al., 2013; Lee et al., 2012; Xu et al., 2012). An important difference between these putative PRC2 substrates and both H3K27 and Jarid2-K116 is the fact that the former are not involved in retro-control loops regulating PRC2 enzymatic activity. Hence, both H3K27me3 and Jarid2-K116me3 are able to bind Eed and promote the allosteric activation of PRC2 enzymatic activity. Such a process is unlikely to occur with other substrates since either the methylated lysine is not preceded by an arginine (Stat3 and Gata4), which we previously showed to be required for PRC2 stimulation (Margueron et al., 2009), or the substrate was only found to be mono-methylated (e.g., RORα).

The discovery that H3K27me3 stimulates PRC2 enzymatic activity and that a single mutation disrupting Eed’s aromatic cage causes a global loss of H3K27me3 in vivo, led to the proposal of a model whereby this positive feedback mechanism might account for the maintenance of this histone mark during cell division (Margueron et al., 2009). In view of this model and of our data, we speculate that Jarid2-methylation could constitute an alternative mechanism to prime PRC2 activity when targeted to chromatin regions devoid of H3K27me3. This would explain why Jarid2 is important while cells engage differentiation but is overall dispensable in undifferentiated ESC, which supposedly mostly maintain H3K27me3 though the positive feedback loop involving H3K27me3 and Eed.

Our results suggest that, in vitro, the methylation of Jarid2 could control the balance between Jarid2me3- and H3K27me3-mediated regulations of PRC2 enzymatic activity due to its higher affinity for Eed. However, it is very likely that in vivo additional histone PTMs modulate the balance between these regulatory loops. Hence, it will be interesting to determine whether H2Aub could, for instance, favor the binding of Eed to H3K27me3 (Kalb et al., 2014). The fact that Jarid2 is dispensable for H3K27me3 deposition in undifferentiated ESC while present at almost all PRC2 targets could be explained by the redundancy between Jarid2-K116me3 and H3K27me3 regulatory loops in this context.

In contrast, upon cell differentiation-induced redistribution of H3K27me3, Jarid2 and its methylation become critical for the appropriate regulation of PRC2 activity. Hence, we observed that in Jarid2 KO and K116A cells H3K27me3 enrichment is lost or reduced during cell differentiation at a subset of PRC2 targets, leaving those genes prone to transcriptional activation. Surprisingly, the losses seem to be compensated by gains of H3K27me3 at unusual genomic location for this mark (e.g., far away from the TSS). Therefore, even though the global level of this mark is unchanged in the Jarid2 mutant cells, the genomic distribution of H3K27me3 is incorrect. The fact that the gains of H3K27me3 retain some selectivity for CpG islands in the Jarid2-K116A, which express Jarid2 with functional DNA-binding domains, suggests that this aberrant targeting is not random but results from the combination of partly functional Jarid2 and other cofactors that misguide PRC2 to new sites. It remains to be determined whether those new H3K27me3 peaks have transcriptional consequences or are constituted of chromatin domains that are somehow acting as a “sink” for the surplus of PRC2 activity. Intriguingly, a similar enrichment for H3K27me3 at intergenic regions was also reported for ESC grown in 2i media (Marks et al., 2012), it would therefore be interesting to investigate whether Jarid2 function could be altered in this context.

In conclusion, Jarid2 protein contains distinct domains that regulate PRC2 function through different mechanisms. Our study provides insights into the molecular mechanisms by which Jarid2 controls PRC2 function during mammalian cell differentiation. Although we now have a deeper understanding of the various functions of the rather unique N-terminal moiety of Jarid2, the role of the C terminus remains unclear. The presence of several conserved domains, such as the enzymatically inactive demethylase-like domain, suggests that this C-terminal portion must have some yet unknown functions. Our study also reveals that Jarid2 and its methylation appear to have a more critical role in a developmental context when compared to undifferentiated cells. Dissecting how the multiple functions of Jarid2 in targeting and modulating PRC2 activity are orchestrated during development remains an exciting challenge for the future.

## Experimental Procedures

Full details on experimental procedures and the list of the used antibodies are available on the Supplemental Experimental Procedures.

### BAC Mutagenesis and Genomic Integration

K116A mutation was generated in the BAC-Jarid2 RP23-152H18 by homologous recombination and counter selection strategy (Bird et al., 2012). The construct was inserted into the genome of Jarid2 KO ESC through transposon-mediated BAC integration as previously described (Rostovskaya et al., 2012).

### Recombinant Proteins Purification

Recombinant hJarid2 fragments 6×His-tag were produced in bacteria. Recombinant mammals and flies PRC2, Aebp2, Jarid2 WT, and K116A were produced in SF9 insect cells as described in Supplemental Experimental Procedures.

### Native Chemical Ligation

Jarid2 109-450 S124C was purified from bacteria and chemically ligated to the C-terminal thioester peptide K116me3 as described in Supplemental Experimental Procedures.

### ΔEed-Jarid2K116me3 Crystal

The ΔEed protein was prepared as previously described (Margueron et al., 2009) and detailed in the Supplemental Experimental Procedures.

### Nanostring Quantification of Dene Expression

Direct digital mRNA analysis of expression was performed using a custom set of oligonucleotides synthetized by NanoString Technologies. Hybridization and analysis were done using the Prep Station and Digital Analyzer purchased from the company.

## Figures and Tables

**Table 1:** Data Collection and Refinement Statistics

		EED-Jarid2
**Data Collection**		

Beamline		Diamond Light Source IO2
Wavelength		0.9795 Å
Space group		P212121
Cell dimensions		
	a, b, c	49.419, 58.144, 127.042 Å
	α, β, γ	90.00°, 90.00°, 90.00°
Resolution (Å)		30–2.0 Å
		(2.09–2.0 Å)
*R*_*sym*_		0.084 (0.365)
*I/ σI*		16.2 (2.23)
Completeness		97.2% (38.2%)
Redundancy		3.9 (1.9)

**Refinement**		

Resolution (Å)		30–2.3 Å
Number of Reflections		16,463
*R*_work_ / *R*_free_		0.174 / 0.224
Number of atoms		
	Protein	2,972
	Solvent	175
*B-*factors		
	Protein	21.15 Å^2^
	Solvent	24.03 Å^2^
RMS deviations		
	Bond lengths	0.007 Å
	Bond Angles	1.074°
Ramachandran plot		
	Favored regions	95.8%
	Allowed region	4.2%
	Outlier region	0.0%

Highest-resolution shell is shown in parentheses.

## Accession Numbers

The GEO repository accession number for the all ChIP-seq datasets reported in this paper is GSE55630. The Protein Data Bank accession number for the structural data reported in this paper is 4X3E.

## Supplemental Information

Document S1. Supplemental Experimental Procedures, Figures S1–S7, and Table S1

Document S2. Article plus Supplemental Information

## References

[bib1] Bartke T., Vermeulen M., Xhemalce B., Robson S.C., Mann M., Kouzarides T. (2010). Nucleosome-interacting proteins regulated by DNA and histone methylation. Cell.

[bib2] Bird A.W., Erler A., Fu J., Hériché J.K., Maresca M., Zhang Y., Hyman A.A., Stewart A.F. (2012). High-efficiency counterselection recombineering for site-directed mutagenesis in bacterial artificial chromosomes. Nat. Methods.

[bib3] Blackledge N.P., Farcas A.M., Kondo T., King H.W., McGouran J.F., Hanssen L.L., Ito S., Cooper S., Kondo K., Koseki Y. (2014). Variant PRC1 complex-dependent H2A ubiquitylation drives PRC2 recruitment and polycomb domain formation. Cell.

[bib4] Brien G.L., Gambero G., O’Connell D.J., Jerman E., Turner S.A., Egan C.M., Dunne E.J., Jurgens M.C., Wynne K., Piao L. (2012). Polycomb PHF19 binds H3K36me3 and recruits PRC2 and demethylase NO66 to embryonic stem cell genes during differentiation. Nat. Struct. Mol. Biol..

[bib5] Brockdorff N. (2013). Noncoding RNA and Polycomb recruitment. RNA.

[bib6] Cai L., Rothbart S.B., Lu R., Xu B., Chen W.Y., Tripathy A., Rockowitz S., Zheng D., Patel D.J., Allis C.D. (2013). An H3K36 methylation-engaging Tudor motif of polycomb-like proteins mediates PRC2 complex targeting. Mol. Cell.

[bib7] Cooper S., Dienstbier M., Hassan R., Schermelleh L., Sharif J., Blackledge N.P., De Marco V., Elderkin S., Koseki H., Klose R. (2014). Targeting polycomb to pericentric heterochromatin in embryonic stem cells reveals a role for H2AK119u1 in PRC2 recruitment. Cell Rep..

[bib8] da Rocha S.T., Boeva V., Escamilla-Del-Arenal M., Ancelin K., Granier C., Matias N.R., Sanulli S., Chow J., Schulz E., Picard C. (2014). Jarid2 Is Implicated in the Initial Xist-Induced Targeting of PRC2 to the Inactive X Chromosome. Mol. Cell.

[bib9] Davidovich C., Zheng L., Goodrich K.J., Cech T.R. (2013). Promiscuous RNA binding by Polycomb repressive complex 2. Nat. Struct. Mol. Biol..

[bib10] He A., Shen X., Ma Q., Cao J., von Gise A., Zhou P., Wang G., Marquez V.E., Orkin S.H., Pu W.T. (2012). PRC2 directly methylates GATA4 and represses its transcriptional activity. Genes Dev..

[bib11] Herranz N., Pasini D., Díaz V.M., Francí C., Gutierrez A., Dave N., Escrivà M., Hernandez-Muñoz I., Di Croce L., Helin K. (2008). Polycomb complex 2 is required for E-cadherin repression by the Snail1 transcription factor. Mol. Cell. Biol..

[bib12] Herz H.M., Mohan M., Garrett A.S., Miller C., Casto D., Zhang Y., Seidel C., Haug J.S., Florens L., Washburn M.P. (2012). Polycomb repressive complex 2-dependent and -independent functions of Jarid2 in transcriptional regulation in Drosophila. Mol. Cell. Biol..

[bib13] Kalb R., Latwiel S., Baymaz H.I., Jansen P.W., Müller C.W., Vermeulen M., Müller J. (2014). Histone H2A monoubiquitination promotes histone H3 methylation in Polycomb repression. Nat. Struct. Mol. Biol..

[bib14] Kaneko S., Son J., Shen S.S., Reinberg D., Bonasio R. (2013). PRC2 binds active promoters and contacts nascent RNAs in embryonic stem cells. Nat. Struct. Mol. Biol..

[bib15] Kaneko S., Bonasio R., Saldaña-Meyer R., Yoshida T., Son J., Nishino K., Umezawa A., Reinberg D. (2014). Interactions between JARID2 and noncoding RNAs regulate PRC2 recruitment to chromatin. Mol. Cell.

[bib16] Kanhere A., Viiri K., Araújo C.C., Rasaiyaah J., Bouwman R.D., Whyte W.A., Pereira C.F., Brookes E., Walker K., Bell G.W. (2010). Short RNAs are transcribed from repressed polycomb target genes and interact with polycomb repressive complex-2. Mol. Cell.

[bib17] Kim T.G., Chen J., Sadoshima J., Lee Y. (2004). Jumonji represses atrial natriuretic factor gene expression by inhibiting transcriptional activities of cardiac transcription factors. Mol. Cell. Biol..

[bib18] Kim H., Kang K., Kim J. (2009). AEBP2 as a potential targeting protein for Polycomb Repression Complex PRC2. Nucleic Acids Res..

[bib19] Kim E., Kim M., Woo D.H., Shin Y., Shin J., Chang N., Oh Y.T., Kim H., Rheey J., Nakano I. (2013). Phosphorylation of EZH2 activates STAT3 signaling via STAT3 methylation and promotes tumorigenicity of glioblastoma stem-like cells. Cancer Cell.

[bib20] Klose R.J., Kallin E.M., Zhang Y. (2006). JmjC-domain-containing proteins and histone demethylation. Nat. Rev. Genet..

[bib21] Klose R.J., Cooper S., Farcas A.M., Blackledge N.P., Brockdorff N. (2013). Chromatin sampling—an emerging perspective on targeting polycomb repressor proteins. PLoS Genet..

[bib22] Landeira D., Fisher A.G. (2011). Inactive yet indispensable: the tale of Jarid2. Trends Cell Biol..

[bib23] Landeira D., Sauer S., Poot R., Dvorkina M., Mazzarella L., Jørgensen H.F., Pereira C.F., Leleu M., Piccolo F.M., Spivakov M. (2010). Jarid2 is a PRC2 component in embryonic stem cells required for multi-lineage differentiation and recruitment of PRC1 and RNA Polymerase II to developmental regulators. Nat. Cell Biol..

[bib24] Lee J.M., Lee J.S., Kim H., Kim K., Park H., Kim J.Y., Lee S.H., Kim I.S., Kim J., Lee M. (2012). EZH2 generates a methyl degron that is recognized by the DCAF1/DDB1/CUL4 E3 ubiquitin ligase complex. Mol. Cell.

[bib25] Li G., Margueron R., Ku M., Chambon P., Bernstein B.E., Reinberg D. (2010). Jarid2 and PRC2, partners in regulating gene expression. Genes Dev..

[bib26] Maenner S., Blaud M., Fouillen L., Savoye A., Marchand V., Dubois A., Sanglier-Cianférani S., Van Dorsselaer A., Clerc P., Avner P. (2010). 2-D structure of the A region of Xist RNA and its implication for PRC2 association. PLoS Biol..

[bib27] Margueron R., Reinberg D. (2011). The Polycomb complex PRC2 and its mark in life. Nature.

[bib28] Margueron R., Justin N., Ohno K., Sharpe M.L., Son J., Drury W.J., Voigt P., Martin S.R., Taylor W.R., De Marco V. (2009). Role of the polycomb protein EED in the propagation of repressive histone marks. Nature.

[bib29] Marks H., Kalkan T., Menafra R., Denissov S., Jones K., Hofemeister H., Nichols J., Kranz A., Stewart A.F., Smith A., Stunnenberg H.G. (2012). The transcriptional and epigenomic foundations of ground state pluripotency. Cell.

[bib30] Musselman C.A., Avvakumov N., Watanabe R., Abraham C.G., Lalonde M.E., Hong Z., Allen C., Roy S., Nuñez J.K., Nickoloff J. (2012). Molecular basis for H3K36me3 recognition by the Tudor domain of PHF1. Nat. Struct. Mol. Biol..

[bib31] Palacios D., Mozzetta C., Consalvi S., Caretti G., Saccone V., Proserpio V., Marquez V.E., Valente S., Mai A., Forcales S.V. (2010). TNF/p38α/polycomb signaling to Pax7 locus in satellite cells links inflammation to the epigenetic control of muscle regeneration. Cell Stem Cell.

[bib32] Pandey R.R., Mondal T., Mohammad F., Enroth S., Redrup L., Komorowski J., Nagano T., Mancini-Dinardo D., Kanduri C. (2008). Kcnq1ot1 antisense noncoding RNA mediates lineage-specific transcriptional silencing through chromatin-level regulation. Mol. Cell.

[bib33] Pasini D., Cloos P.A., Walfridsson J., Olsson L., Bukowski J.P., Johansen J.V., Bak M., Tommerup N., Rappsilber J., Helin K. (2010). JARID2 regulates binding of the Polycomb repressive complex 2 to target genes in ES cells. Nature.

[bib34] Peng J.C., Valouev A., Swigut T., Zhang J., Zhao Y., Sidow A., Wysocka J. (2009). Jarid2/Jumonji coordinates control of PRC2 enzymatic activity and target gene occupancy in pluripotent cells. Cell.

[bib35] Pengelly A.R., Copur Ö., Jäckle H., Herzig A., Müller J. (2013). A histone mutant reproduces the phenotype caused by loss of histone-modifying factor Polycomb. Science.

[bib36] Qin S., Guo Y., Xu C., Bian C., Fu M., Gong S., Min J. (2013). Tudor domains of the PRC2 components PHF1 and PHF19 selectively bind to histone H3K36me3. Biochem. Biophys. Res. Commun..

[bib37] Redrup L., Branco M.R., Perdeaux E.R., Krueger C., Lewis A., Santos F., Nagano T., Cobb B.S., Fraser P., Reik W. (2009). The long noncoding RNA Kcnq1ot1 organises a lineage-specific nuclear domain for epigenetic gene silencing. Development.

[bib38] Rinn J.L., Kertesz M., Wang J.K., Squazzo S.L., Xu X., Brugmann S.A., Goodnough L.H., Helms J.A., Farnham P.J., Segal E., Chang H.Y. (2007). Functional demarcation of active and silent chromatin domains in human HOX loci by noncoding RNAs. Cell.

[bib39] Rostovskaya M., Fu J., Obst M., Baer I., Weidlich S., Wang H., Smith A.J., Anastassiadis K., Stewart A.F. (2012). Transposon-mediated BAC transgenesis in human ES cells. Nucleic Acids Res..

[bib40] Schmitges F.W., Prusty A.B., Faty M., Stützer A., Lingaraju G.M., Aiwazian J., Sack R., Hess D., Li L., Zhou S. (2011). Histone methylation by PRC2 is inhibited by active chromatin marks. Mol. Cell.

[bib41] Shen X., Kim W., Fujiwara Y., Simon M.D., Liu Y., Mysliwiec M.R., Yuan G.C., Lee Y., Orkin S.H. (2009). Jumonji modulates polycomb activity and self-renewal versus differentiation of stem cells. Cell.

[bib42] Simon J.A., Kingston R.E. (2013). Occupying chromatin: Polycomb mechanisms for getting to genomic targets, stopping transcriptional traffic, and staying put. Mol. Cell.

[bib43] Son J., Shen S.S., Margueron R., Reinberg D. (2013). Nucleosome-binding activities within JARID2 and EZH1 regulate the function of PRC2 on chromatin. Genes Dev..

[bib44] Voigt P., LeRoy G., Drury W.J., Zee B.M., Son J., Beck D.B., Young N.L., Garcia B.A., Reinberg D. (2012). Asymmetrically modified nucleosomes. Cell.

[bib45] Voigt P., Tee W.W., Reinberg D. (2013). A double take on bivalent promoters. Genes Dev..

[bib46] Woo C.J., Kharchenko P.V., Daheron L., Park P.J., Kingston R.E. (2010). A region of the human HOXD cluster that confers polycomb-group responsiveness. Cell.

[bib47] Woo C.J., Kharchenko P.V., Daheron L., Park P.J., Kingston R.E. (2013). Variable requirements for DNA-binding proteins at polycomb-dependent repressive regions in human HOX clusters. Mol. Cell. Biol..

[bib48] Xu K., Wu Z.J., Groner A.C., He H.H., Cai C., Lis R.T., Wu X., Stack E.C., Loda M., Liu T. (2012). EZH2 oncogenic activity in castration-resistant prostate cancer cells is Polycomb-independent. Science.

[bib49] Yuan W., Xu M., Huang C., Liu N., Chen S., Zhu B. (2011). H3K36 methylation antagonizes PRC2-mediated H3K27 methylation. J. Biol. Chem..

[bib50] Yuan W., Wu T., Fu H., Dai C., Wu H., Liu N., Li X., Xu M., Zhang Z., Niu T. (2012). Dense chromatin activates Polycomb repressive complex 2 to regulate H3 lysine 27 methylation. Science.

[bib51] Zhang Z., Jones A., Sun C.W., Li C., Chang C.W., Joo H.Y., Dai Q., Mysliwiec M.R., Wu L.C., Guo Y. (2011). PRC2 complexes with JARID2, MTF2, and esPRC2p48 in ES cells to modulate ES cell pluripotency and somatic cell reprogramming. Stem Cells.

[bib52] Zhao J., Sun B.K., Erwin J.A., Song J.J., Lee J.T. (2008). Polycomb proteins targeted by a short repeat RNA to the mouse X chromosome. Science.

