# Supplementary material for: Jarid2 Methylation via the PRC2 Complex Regulates H3K27me3 Deposition during Cell Differentiation
Source: Mol Cell. 2015 Mar 5;57(5):769–83. doi: 10.1016/j.molcel.2014.12.020 (PMC4352895; doi:10.1016/j.molcel.2014.12.020)
Supplement: Document S1. Supplemental Experimental Procedures, Figures S1–S7, and Table S1 [file mmc1.pdf]

Molecular Cell
Supplemental Information

Jarid2 Methylation via the PRC2 Complex Regulates H3K27me3 Deposition during Cell Differentiation

Serena Sanulli, Neil Justin, Aurélie Teissandier, Katia Ancelin, Manuela Portoso, Matthieu Caron, Audrey Michaud, Berangère Lombard, Simao T. da Rocha, John Offer, Damarys Loew, Nicolas Servant, Michel Wassef, Fabienne Burlina, Steve J. Gamblin, Edith Heard, and Raphaël Margueron

1) Supplemental Figures and Legends

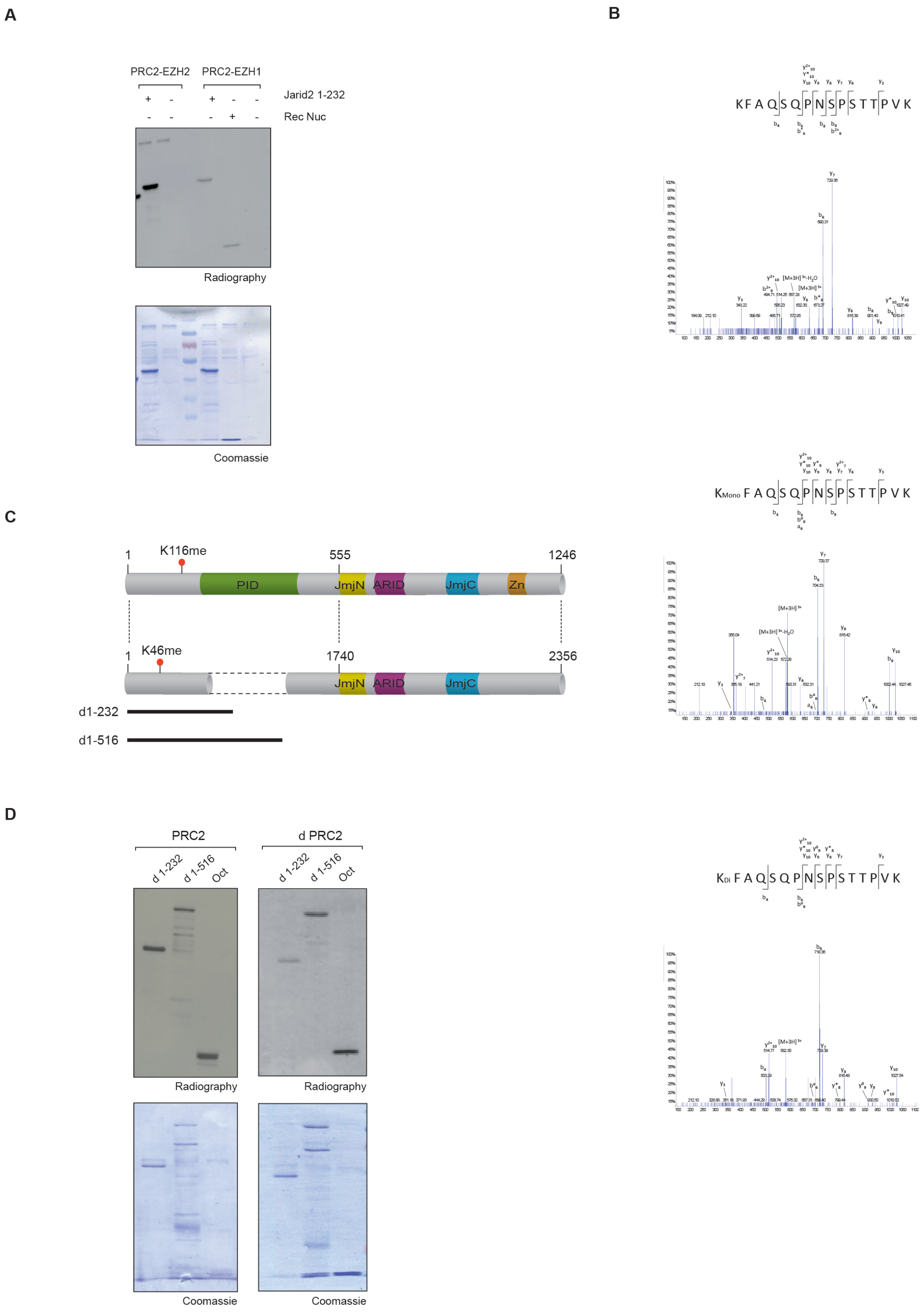

Supplemental Figure 1 (Related to Fig 1)

(A) KMT assay using PRC2-Ezh1 or PRC2-Ezh2 as enzyme and Jarid2 fragment aa 1-232 as substrate. (B) MS/MS spectrum of the peptide precursor ion at m/z 572.97, 577.64 and 582.31 determining unmodified, mono- and di-methylated Lysine 116 in the peptide ^{116}KFAQSQPNSPSTTPVK^{131} of Jarid2 fragment 1-232. (C) Schematic representation of human (top) and *Drosophila* (bottom) Jarid2 proteins. dJarid2 fragments used in (D) are shown. (D) KMT assay using mammalian PRC2 or dPRC2 as enzyme and dJarid2 aa fragments 1-232 or 1-516 as substrates, octamers (Oct) are used as positive control.

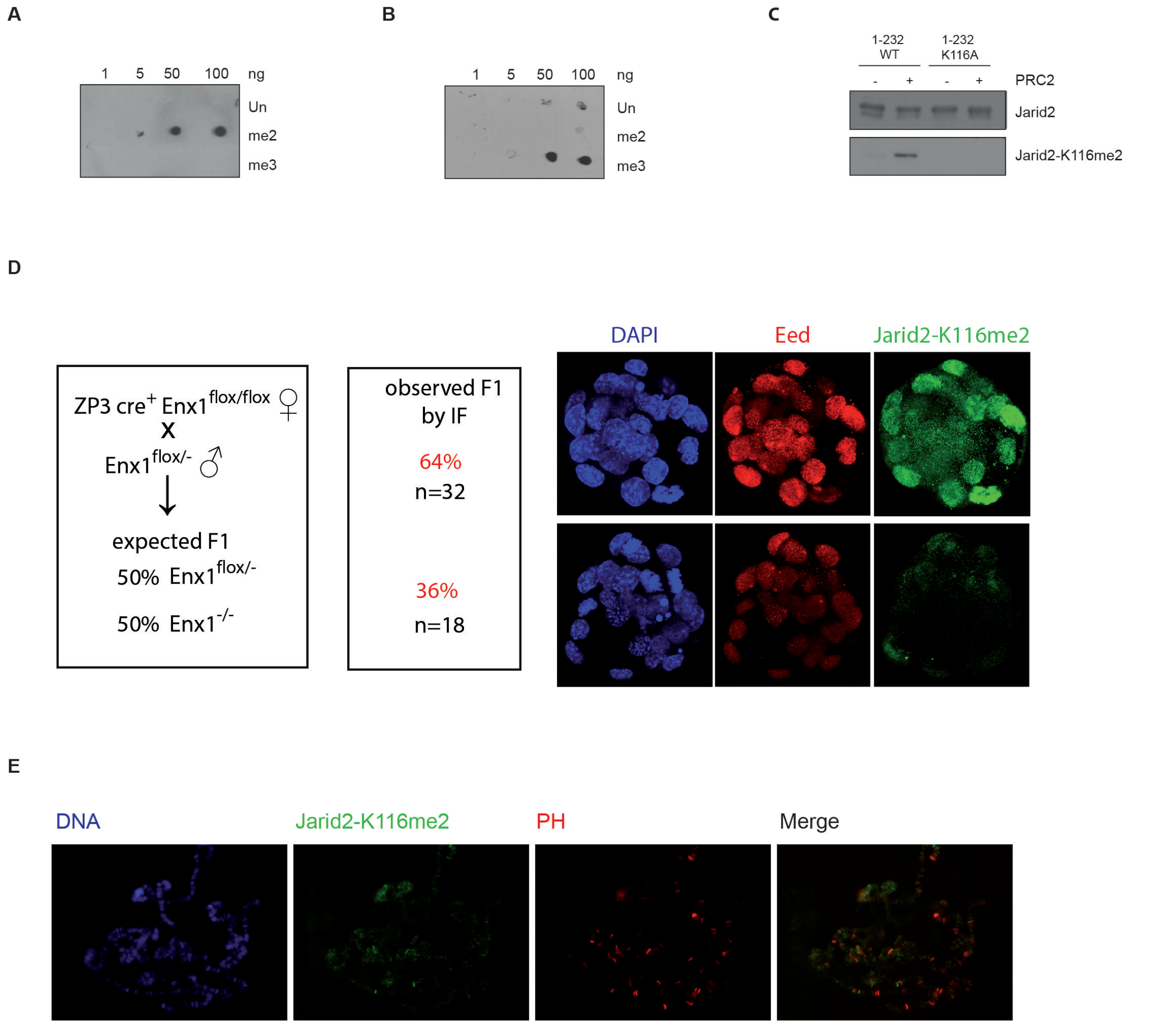

Supplemental Figure 2 (Related to Fig 2)

(A and B) Dot blots showing the specificity of the Jarid2-K116me2 (A) and Jarid2-K116me3 (B) antibodies. The amounts of peptides correspond to Jarid2-K116 un- (Un), di- (me2) and tri- (me3) methylated peptides are indicated on top. (C) KMT assay using PRC2 as enzyme and Jarid2 fragment 1-232 WT or K116A mutant as substrates, followed by WB with the indicated antibodies, showing the specificity of the antibodies on recombinant methylated proteins. (D) IF staining of Jarid2-K116me2 in mouse blastocyst expected to be either Ezh2 f/- or Ezh2 -/- (maternal deletion using the Zp3-Cre). Nuclei are stained with DAPI. Embryos were harvested at 16 cell-stage. Ezh2 -/- embryos appeared mildly delayed. (E) *Drosophila* polytene chromosome staining performed on WT flies with the indicated antibodies.

A

	Jarid2 total	Jarid2-K116me2	Input
N. of sequenced reads	45 474 408	41 207 017	38 967 462
N. of mapped reads (in%)	44 120 208 (97.05%)	39 933 396 (96.91%)	38 075 960 (97.71%)
N. reads after duplicates removal	37 269 772 (84.47%)	33 330 697 (83.46%)	32 299 590 (84.82%)

B

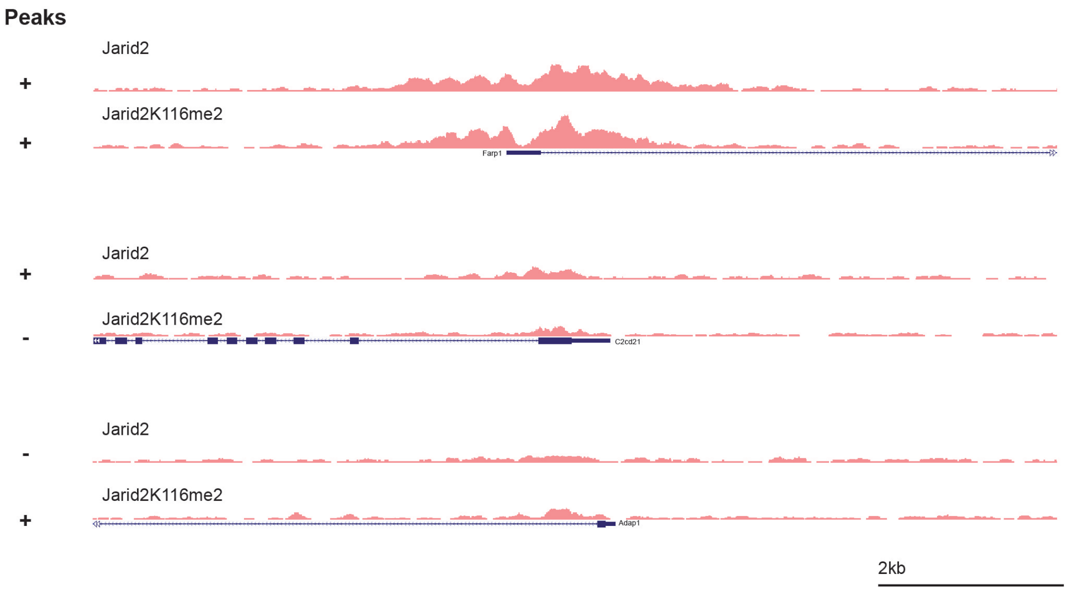

C

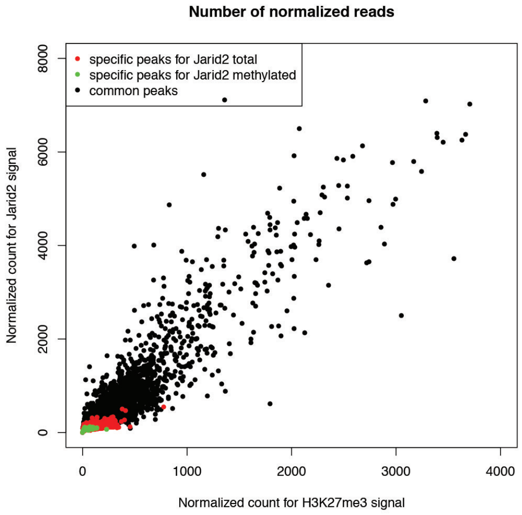

Supplemental Figure 3 (Related to Fig 3)

(A) Number of sequenced and mapped reads in the Jarid2 and Jarid2-K116me2 ChIP-seq experiments. (B) Snapshots of representative peaks common to total Jarid2 and Jarid2-K116me2 ChIP-Seq or specific for one of the two as indicated on the left. (C) Normalized read

counts for Jarid2 *vs* H3K27me3 ChIP-seq at total and methylated Jarid2 target peaks. Read counts for H3K27 were analyzed after extending Jarid2 peaks of 0,5kb on each side. Total Jarid2 ChIP-seq described in figure 3. H3K27me3 ChIP-seq (rescued Jarid2 WT cell) described in figure 7.

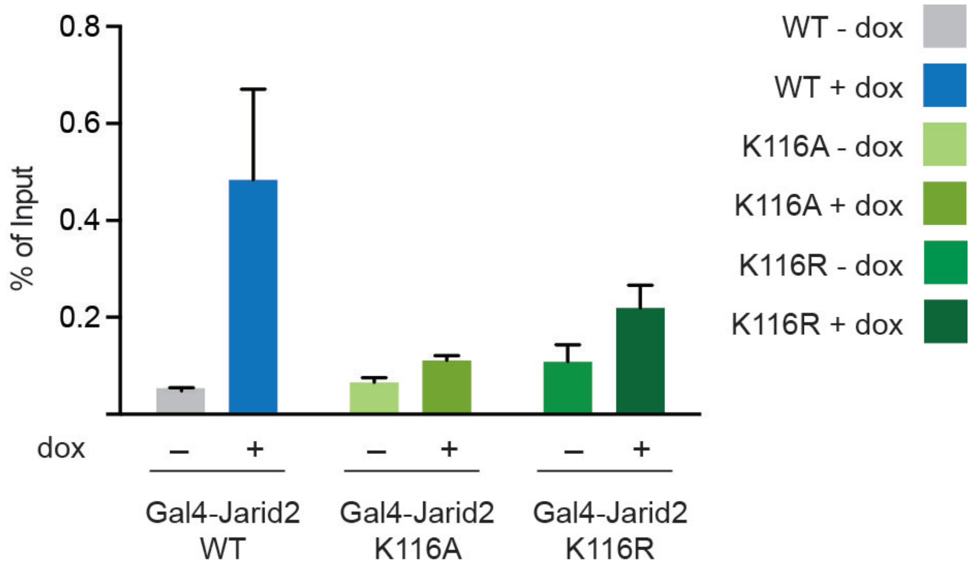

Supplemental Figure 4 (Related to Fig 4)

ChIP performed on T-rex 293 Gal4-Jarid2 WT, K116A and K116R cell lines. H3K27me2/3 (7B11G5) antibody was used for ChIP. Y-axis represents percent of input (mean ±SD, n≥2).

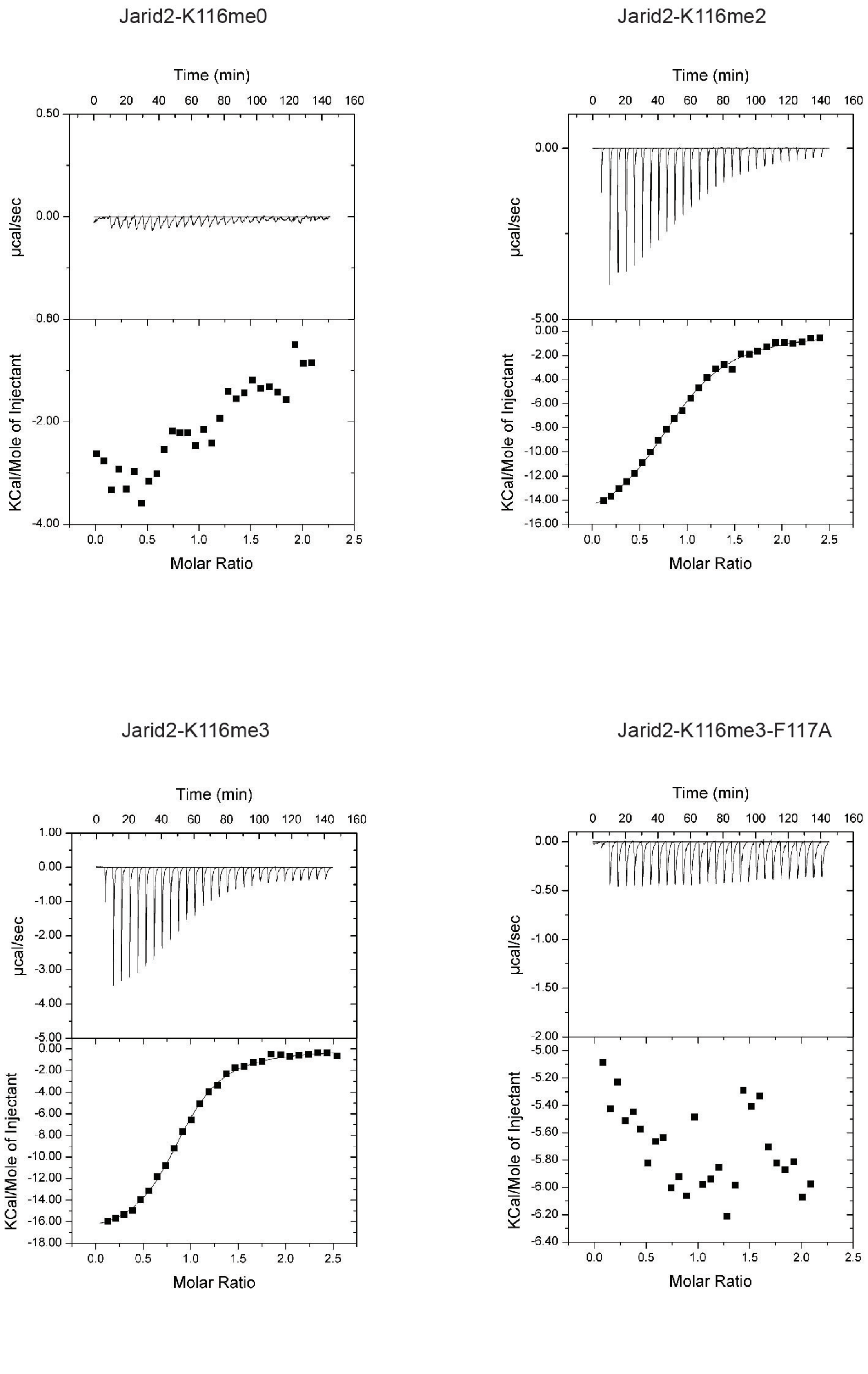

Supplemental Figure 5 (Related to Fig 5)

ITC curves for Eed binding to the indicated peptides.

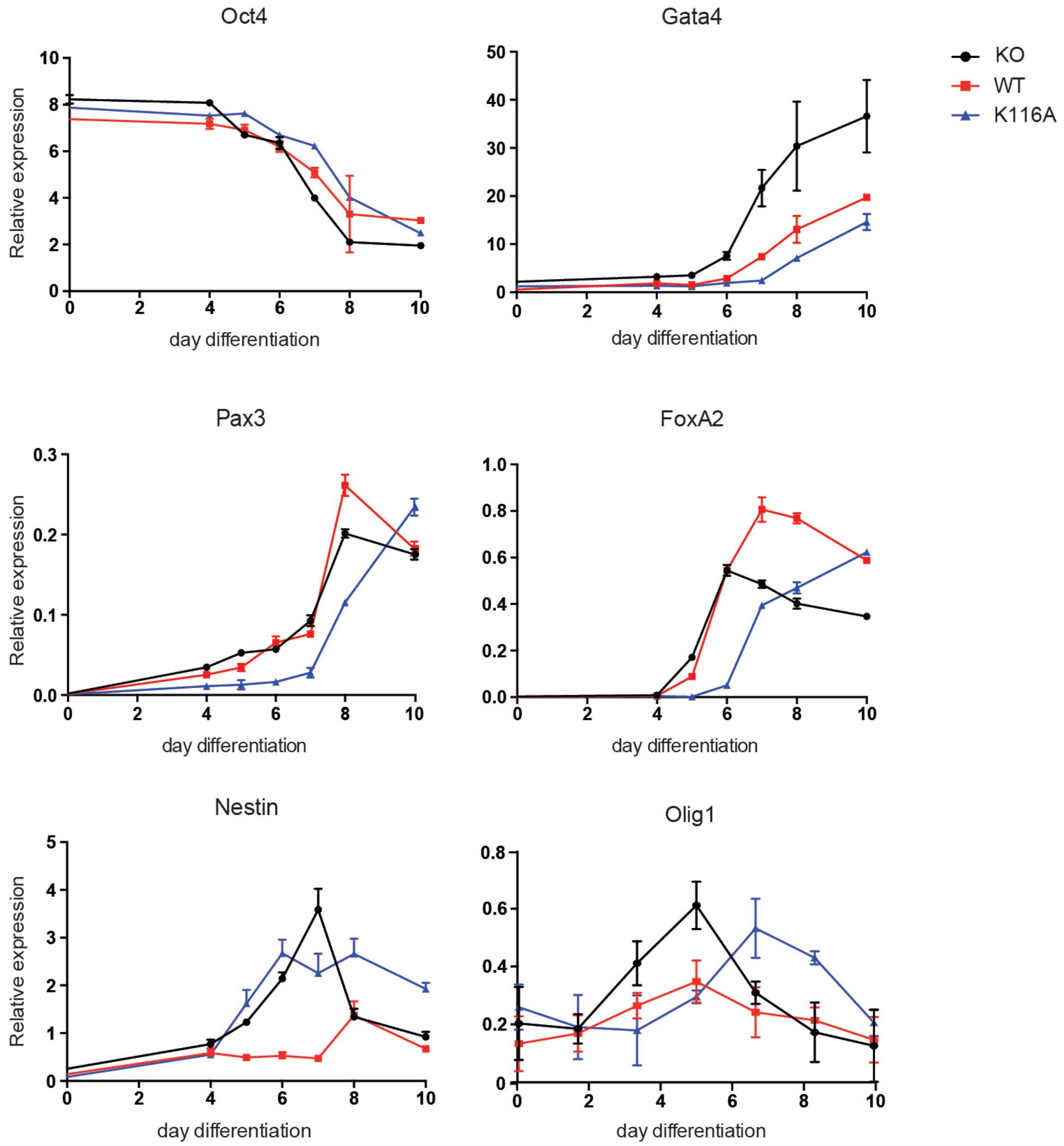

Supplemental Figure 6 (Related to Fig 6)

Messenger RNA (mRNA) levels were quantified by RT-qPCR upon differentiation of ES into EB. Values are normalized to TBP mRNA and plotted as $2^{-\Delta Ct}$ (mean ±SD, n≥2).

A

	INPUT DNA	ES KO 1	ES KO 2	ES WT 1	ES WT 2	ES K116A 1	ES K116A 2
Number of sequenced reads	24 473 481	37 226 259	30 922 074	45 301 618	34 207 213	37 046 449	37 673 884
Number of mapped reads (in %)	23 936 060 (97.80%)	36 389 538 (97.75%)	30 289 660 (97.95%)	44 379 213 (97.96%)	33 473 130 (97.85%)	36 244 797 (97.84%)	36 811 247 (97.71%)
Number of reads after removed duplicates	19 153 409 (80,02%)	32 768 745 (90,05%)	27 987 240 (92,4%)	40 698 850 (91,70%)	30 906 033 (92,33%)	33 031 511 (91,13%)	33 686 455 (91,51%)
Fragment length (from Bioanalyzer)	274bp	331bp	338bp	285bp	318bp	270bp	308bp

		EB KO 1	EB KO 2	EB WT 1	EB WT 2	EB K116A 1	EB K116A 2
Number of sequenced reads		40 834 363	52 059 975	28 162 928	26 575 175	37 277 365	62 108 749
Number of mapped reads (in %)		40 010 529 (97.98%)	51 039 232 (98.04%)	27 560 912 (97.86%)	25 997 552 (97.83%)	36 460 560 (97.81%)	60 767 850 (97.84%)
Number of reads after removed duplicates		35 328 363 (88,3%)	45 461 882 (89,07%)	25 088 395 (91,03%)	23 887 572 (91,88%)	32 921 572 (90,29%)	53 790 640 (88,52%)
Fragment length (from Bioanalyzer)		327bp	364bp	342bp	364bp	322bp	352bp

B

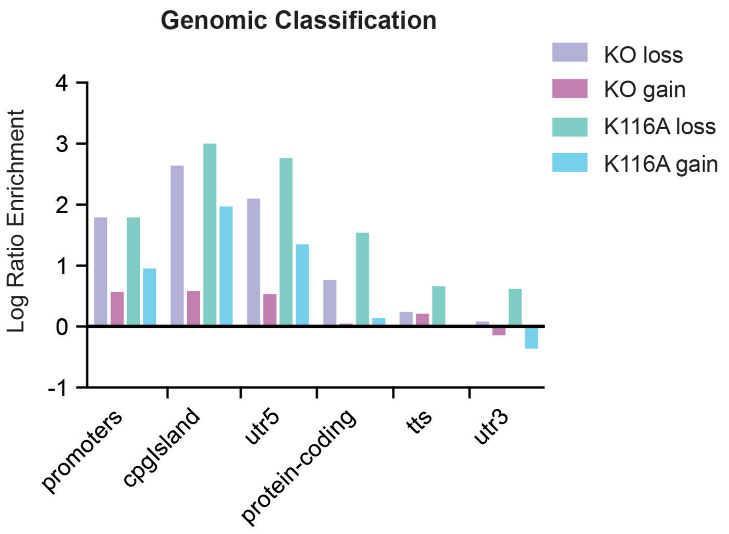

Supplemental Figure 7 (Related to Fig 7)

(A) Number of sequenced and mapped reads in the H3K27me3 ChIP-seq experiments (3 cell lines both undifferentiated "ESC" or differentiated "EB"). (B) Genomic ontology of peaks gaining or loosing H3K27me3 during cell differentiation specifically in the Jarid2 KO or Jarid2 K116A cells.

2) Supplemental Table and Legend

	ES KO 1	ES KO 2	ES WT 1	ES WT 2	ES K116A 1	ES K116A 2	EB KO 1	EB KO 2	EB WT 1	EB WT 2	EB K116A 1	EB K116A 2
ES KO 1	1	0,985	0,97	0,989	0,975	0,979	0,929	0,904	0,965	0,972	0,876	0,861
ES KO 2	0,985	1	0,928	0,993	0,953	0,978	0,932	0,912	0,964	0,97	0,881	0,872
ES WT 1	0,97	0,928	1	0,953	0,969	0,948	0,887	0,862	0,942	0,95	0,85	0,833
ES WT 2	0,989	0,993	0,953	1	0,96	0,978	0,922	0,9	0,97	0,978	0,877	0,865
ES K116A 1	0,975	0,953	0,969	0,96	1	0,99	0,918	0,897	0,953	0,956	0,917	0,905
ES K116A 2	0,979	0,978	0,948	0,978	0,99	1	0,931	0,912	0,965	0,968	0,928	0,919
EB KO 1	0,929	0,932	0,887	0,922	0,918	0,931	1	0,996	0,971	0,967	0,933	0,929
EB KO 2	0,904	0,912	0,862	0,9	0,897	0,912	0,996	1	0,957	0,953	0,925	0,927
EB WT 1	0,965	0,964	0,942	0,97	0,953	0,965	0,971	0,957	1	0,996	0,936	0,927
EB WT 2	0,972	0,97	0,95	0,978	0,956	0,968	0,967	0,953	0,996	1	0,923	0,914
EB K116A 1	0,876	0,881	0,85	0,877	0,917	0,928	0,933	0,925	0,936	0,923	1	0,995
EB K116A 2	0,861	0,872	0,833	0,865	0,905	0,919	0,929	0,927	0,927	0,914	0,995	1

Supplementary Table 1 (Related to Fig 7)

Pearson correlation between each ChIP-seq samples. 1 and 2 refer to biological replicates**.**

3) Supplemental Experimental procedures

Cloning

dJarid2 clone LD17709 was obtained from the Drosophila Genomics Resource Center and the fragments 1-232 and 1-516 were cloned in pet102 for bacterial expression.
hJarid2 fragments were cloned in pET102 vector (Invitrogen) for bacterial expression.

Site directed mutagenesis

Lysine 116 was mutated to Alanine and Arginine following the QuikChange Site-Directed Mutagenesis Kit (Stratagene).

Cell lines

T-Rex 293 cells (Invitrogen) were grown according to the manufacturer's instructions. First, 5XGal4RE-tk-Luc-Neo plasmid was stably integrated into the cells and selected by G418. Subsequently, the selected clone was generated by stable transfection of pCDNA4-T0-Gal4-hJarid2 WT and K116A. Single clones are selected with zeocin (300 μg/ml) and screened for the expression of Gal4-Jarid2 48 hours after doxycycline induction at 1μg/ml (Sigma). Stable clones of 293T cell over-expressing pCMV4-HA-Flag-hJarid2 WT or K116A and resistant to G418 were screened for the expression of the Flag-tagged proteins by WB.
ESC were cultured on gelatin-coated dishes in DMEM media supplemented with 15% FBS, 100mM non essential amino acids, 0.1mM 2-mercaptoethanol, 1mM L-Glutamine (Invitrogen) and 103U/ml leukemia inhibitory factor (LIF) (ESG1107, Millipore).
ESC E14 and Eed-/- were cultured as previously described (Li et al., 2010). ΔEzh2 f/f ROSA Cre-ERT2 ESC were generated in E.H. laboratory. Jarid2-/- ESC were generously provided by S. Orkin.
D. melanogaster S2 cells were cultured in Schneider's Drosophila medium (Invitrogen) supplemented with 10% FBS at 25 °C.

ESC differentiation

EBs are formed by hanging drop method. Cells are diluted at 1000 cells/20μl drop in ESC media in absence of LIF. After 2 days EBs are flushed in low adherence plates.

shRNA

ShRNA were designed against hJarid2 accordingly to

http://www.broadinstitute.org/rnai/public/seq/search. Oligonucleotides were annealed and sub-cloned in the pLKO.1 vector (Addgene). Cells were infected and selected with 2μg/ml puromycin.

Luciferase Assay

Luciferase reporter activities were measured in whole cell lysates using the Luciferase Assay System (Promega, #E15020) and Fluostar Optima BMG labtech luminometer. All experiments were done in biological and technical triplicates and normalized for protein concentration (Bradford).

Antibodies

WB, ChIP and IF were performed using the following antibodies: polyclonal antibodies specific to Jarid2-K116 me2 and me3 were raised against a synthetic peptide H2N-CRLQAQRK(dimethyl)FAQSQ-CONH2 and CRLQAQRK(trimethyl)FAQSQ-CONH2 representing aa 109-121. The serum was first counter-selected on a column containing the unmodified peptide and subsequently affinity purified on the methylated peptide. Antibodies against Ezh2, Suz12, Jarid2 and H3K27me2/3 were previously described (Margueron et al., 2008); total H3 (39163) and H3K4me3 (39159) were purchased from Active Motif; Gal4 (06-262) for ChIP was purchased from Millipore; Gal4 for WB from Santa Cruz Biotechnologies (sc-510); H3K27me3 (ab6002) and Lamin B1 (ab16048) were purchased from Abcam; Flag M2 was purchased from Sigma (F1804); Oct4 (BD 611203) BD Biosciences. Eed antibody (M26) was kindly provided by A. Otte to E. H. Anti d-Jarid2 antibody was kindly provided by M. Yamaguchi and used 1:1000 for WB and 1:200 for polytene staining. Anti Ph antibody was kindly provided by G. Cavalli.

Nuclear Extract and Immunoprecipitation

For nuclear extract preparation cells were incubated with buffer A (10mM Hepes pH 7.9, 2.5mM $MgCl_2$, 0.25M sucrose, 0.1% NP40, 0.5mM DTT, 1mM PSMF) for 10 min on ice, centrifuged at 8000 rpm for 10 min, resuspended in buffer B (25mM Hepes pH 7.9, 1.5mM $MgCl_2$, 700 mM NaCl, 0.5mM DTT, 0.1 mM EDTA, 20% glycerol), sonicated and centrifuged at 14000 rpm 15min.

For immunoprecipitation 1mg of nuclear extract was incubated with 1-3μgr of antibody bound to protein A/G overnight. Then beads were washed three times with BC300 (50mM Tris pH7.9, 300mM KCl, 2mM EDTA, 10% Glycerol, and protease inhibitors), and eluted with

0.2 M glycine pH 2.6. For co-immunoprecipitation, samples were first dialyzed against BC250 (50mM Tris pH7.9, 250mM KCl, 2mM EDTA, 10% Glycerol, and protease inhibitors), and then immunoprecipitated.

Protein extract fractionation

Cells are resuspended in buffer A_1 (10mM HEPES pH 7.9, 10mM KCl, 1.5mM $MgCl_2$, 0.34M sucrose, 10% glycerol, 0.1% Triton-X 100, 1mM DTT), spin 5min 2000rpm to obtain the cytoplasm fraction. Nuclei are lysed in buffer B_1 (3mM EDTA, 0.2mM EGTA, 1mM DTT) for 30 min in rotation and spin 5min at 2500rpm to obtain the nuclear fraction. Pellets are resuspended in buffer C (10mM Pipes pH 6.8, 1mM $CaCl_2$, 50mM NaCl) in presence of 2U of MNase and incubated at 37°C in shaking for 15 min. DNA digestion is stopped with EGTA 1mM final. Centrifugation 5min at 2500rpm separates chromatin soluble (supernatant) and chromatin insoluble (pellet) fractions. The pellet was solubilized in buffer C complemented with NaCl 1M final concentration and sonicated. All the buffers are supplement with protease inhibitors.

Dot Blot

1, 5, 20, 100 ng of peptides were spotted on nitrocellulose membrane and let dry for 30 min. After 20 min blocking in 5% milk T-TBS, primary antibodies are incubated in 1% BSA Tween 0,5%-TBS for 30 min at RT, followed by secondary antibodies for 30 min at RT.

Cell lines

First, 5XGal4RE-tk-Luc-Neo plasmid was stably integrated into the T-Rex 293 cells (Invitrogen) and selected by puromycin (1 μg/ml). Subsequently, the selected clone was generated by stable transfection of pCDNA4-T0-Gal4-hJarid2 WT and K116A. Single clones are selected with zeocin (300 μg/ml) and screened for the expression of Gal4-Jarid2 48 hours after doxycycline induction at 1μg/ml (Sigma).

Stable clones of 293T cell over-expressing pCMV4-HA-Flag-hJarid2 WT or K116A and resistant to G418 were screened for the expression of the Flag-tagged proteins by WB.

Recombinant proteins purification

Recombinant hJarid2 fragments contain a 6XHis-tag and were produced in bacteria using the pET102 system. His-tagged proteins were purified on Ni-NTA beads in His buffer (350mM

NaCl, 0.5% NP40, 15% Glycerol, 10mM HEPES pH 7.6) and protease inhibitors. Elutions were performed in His Buffer plus 200mM Imidazole.
Recombinant mammals and flies PRC2, Aebp2, full length Jarid2 WT and K116A were Flag-tagged and all produced in SF9 insect cells after infection with the corresponding baculoviruses as described previously (Li et al., 2010; Margueron et al., 2009; Margueron et al., 2008). Lysates containing Flag-tagged proteins were resuspended in BC300, sonicated and clarified by centrifugation before incubation with Flag-beads (M2-beads) and eluted with Flag peptide.

Baculoviruses production

hJarid2 WT and K116A baculoviruses were produced accordingly to Bac-to-Bac Baculovirus Expression Systems (Invitrogen) starting from pFASTbac vectors.

Native chemical ligation

Sumo-Jarid2 109-450 N123G/S124C, N123G/S124C/K116A, or 124-450 S124C were cloned in pGEX-5X vectors (GE), expressed in bacteria, digested with sumo-protease and purified on SP-HP column (GE). Jarid2 124-450 S124C was purified by HPLC on a RP-C18 column (22 x 250 mm, Vydac) using the gradient 0 to 55% B in A over 30 min with a flow rate of 15 $mL.min^{-1}$ (A = 0.1 % CF_3COOH in H_2O, B = 0.1 % CF_3COOH in CH_3CN). Synthetic peptides were purchased from CS-Bio.
Jarid2 124-450 S124C (1.35 mg, 37 nmol) and the C-terminal thioester peptide K116me3 (1 equiv.) were dissolved in degassed ligation buffer (200 mM sodium phosphate, 6 M Guanidine, 2 mM EDTA, 50 mM tris(2-carboxyethyl)phosphine, 60 mM 4-mercaptophenylacetic acid, pH 7) to give a final concentration for each fragment of 0.5 mM. The reaction mixture was heated at 40 °C and monitored by analytical HPLC on a diPhenyl column (4.6 x 150 mm, Vydac) using the gradient 0 to 45 % B in A over 40 min with a flow rate of 1 $mL.min^{-1}$. The reaction was completed in 5 hours and the ligation product Jarid2 K116me3 was purified by HPLC and characterized by MALDI-TOF MS. Lyophilized proteins were resuspended in 7M urea and dialyzed against BC250 containing protease inhibitors.

KMT assay

KMT assay were performed as described previously (Margueron et al., 2009). Briefly, the reaction containing 200ng of PRC2-Aebp2, 1 μg of substrates, 4mM DTT was incubated in methylation reaction buffer (50mM Tris-HCl pH 8.5, 2.5mM $MgCl_2$) in presence of ^3H-SAM at 30°C for 15 min or 30 min.

Reactions were stopped by boiling 5 min in SDS Laemmli buffer, run on acrylamide gels and transferred on PVDF membranes. When added to the reaction, peptides are at 10-50μM concentrations. Nucleosomes were generated by salt dialysis. H3K27me3 nucleosomes were generated as described in Voigt et al., 2013.

Mass spectrometry analysis

Cold-KMT assay was performed as described above in the presence of PRC2 on the recombinant Jarid2 1-232 fragment with 50μM cold SAM (Sigma). The reaction was stopped by boiling 5 min in SDS Laemmli buffer and run on acrylamide gel. After Coomassie coloration, the band was cut out, washed and proteins were reduced with 10 mM DTT prior to alkylation with 55 μM Iodoacetamide. After washing and shrinking of the gel pieces with 100% acetonitrile, in-gel digestion was performed using trypsin overnight in 25 mM ammonium bicarbonate at 30°C. The extracted peptides were analysed by nano-LC-MS/MS using an Ultimate3000 system (Dionex S.A.) coupled to a QSTAR Elite mass spectrometer (Applied Biosystems/MDS SCIEX). Samples are loaded on a C18 precolumn (300 μm inner diameter x 5 mm; C18 PepMap™ guard column, Dionex S. A.) at 20 μl/min in 100% solvent A (2% acetonitrile, 0.1% acid formic). After 3 min of desalting, the precolumn was switched on line with the analytical C18 column (75 μm inner diameter x 50 cm; C18 PepMap™, Dionex S. A.) equilibrated in 95% solvent A. Bound peptides were eluted using a 60 min linear gradient (from 5% to 50% (v/v)) of solvent B (80% acetonitrile, 0.085% formic acid) at a 200 nl/min flow rate. TOF-MS survey scan was acquired for 1 s over a mass range of 400-1200 m/z. An information-dependent acquisition method was used to acquire product ion scans on the three most intense ions per cycle over a mass range of 65–2000 m/z, excluding previously gated ions for 60s.

The resulting spectra were then analyzed using the Mascot™ search engine against an in-house database (Jarid2 fragment 1-232) and the SwissProt Mus. musculus (house mouse) Protein Database (2013 06 03, 16620 sequences). For all experiments, a precursor ion mass tolerance of 0.2 Da was applied allowing up to 3 miss-cleavages. The fragment ion mass tolerance was set to 0.2 Da. Protein N-terminal acetylation, carbamidomethylation of cysteines, oxidation of methionine and methylation, dimethylation and trimethylation of Lysines were set as variable modifications and no fixed modifications were set. All peptide matches were validated in myProMS (Poullet et al., 2007) with the estimated false discovery rate (FDR) less than 1%.

ΔEed-Jarid2K116me3 crystal

The ΔEed protein was prepared as previously described (Margueron et al., 2009). For the crystallization trials, protein solutions were prepared as a ΔEed complex solution at 1.5 mg ml^{-1} with peptide at a sevenfold higher molar ratio. All protein solutions contained TCEP at 15 mM concentration. Crystals were grown at 18 °C using the vapour diffusion technique in hanging drops. Drops were prepared by mixing equal volumes of ΔEed protein complex with reservoir solution containing 3.7–3.9 M formate solution. Crystals were transferred into mother liquor with 5–10% glycerol before flash cooling in liquid nitrogen. Diffraction data for the Jarid2-K116me3 and me2 protein complex crystals were collected using an in-house MicroMax 007HF rotating anode coupled to a $RaxisIV^{++}$ detector at Diamond Light Source on Beamline IO2 at a wavelength of 0.9795 Angstrom. Data were integrated using Denzo and scaled with Scalepack. The protein complex crystal structures were solved by molecular replacement using PHENIX and the previously published ΔEed structure as the search model. Standard refinement was carried out with refmac5 together with manual model building with Coot. Figures were created with Pymol (DeLano Scientific; http://pymol.sourceforge.net/).

Alkaline Phosphatase and Colony formation assay

Alkaline Phosphatase staining was performed with the Stamgent Alkaline Phosphatase Staining Kit (Milteny Biotec SAS). For colony formation assay, 100 or 500 cells were plated on gelatin-coated 6 well plates and let grown for 7 days, before staining with methylene blue solution (0.2% methylene blue in 70% ethanol) and washes with 70% ethanol.

Cell growth assay

50000 cells were plated in 6 well dishes in triplicated and counted every 24 hours over 4 days.

ESC immunofluorescence

Cells are grown on coverslips, fixed 4% paraformaldehyde 5min at RT, permeabilized with 0.5% Triton-X 100 in PBS 5 min RT, blocked with 20% goat serum in PBS and incubated overnight with primary antibody: Oct4 1:500, Jarid2 1:500, Ezh2 1:500, Jarid2-K116me2 1:750. Alexa Fluor Dyes secondary antibodies (Invitrogen) are used 1:500.

Mouse embryo collection and immunostaining

All animals used in the studies were handled with care and experiments were done according to the guidelines from French legislation and institutional policies. Preimplantation embryos were obtained from superovulated female mice (4-to-8-weeks-old). They were collected in M2

medium (Sigma) by flushing the uterus at 68h (for 16-cell stage) or 92h (for blastocyst) after hCG (human chorionic gonadotropin) injection. Wild-type embryos were obtained from B6D2F1 intercrosses.

Maternal and paternal Ezh2-deficient embryos were generated from mating between Ezh2 $^{Flox/Flox}$ Zp3 cre $^{+/-}$ females with Ezh2 $^{KO/+}$ or $^{KO/Flox}$ males. Immunofluorescence was carried out as described previously (Torres-Padilla et al. 2006), with some modifications. After removal of the zona pellucida with acid Tyrode's solution (Sigma), embryos were fixed in 4% paraformaldehyde, 0,2% sucrose, 0.04% Triton-X100 and 0.3% Tween20 in PBS for 15 min at 37°C. After permeabilization with 0.5% Triton-X100 in PBS for 30 minutes at room temperature, embryos were washed in PBStp (0.05% Triton-X100; 1mg/ml polyvinyl pyrrolidone (PVP-Sigma)) then blocked and incubated with anti Jarid2-K116me2 (1/400) and anti Eed (1/100; M26) antibodies in 1% BSA, 0.05% Triton-X100 for ~16h at 4°C. Embryos were washed in PBStp twice and blocked 30 minutes in 1% BSA in PBStp and incubated for 2h with Alexa conjugated anti rabbit or anti mouse antibodies (Invitrogen/Molecular probes) at room temperature. After washing, embryos were mounted in Vectashield (Clinisciences) containing DAPI for visualizing the DNA. Image were acquired on a Zeiss LSM700 inverted confocal microscope with a Plan apo DICII (numerical aperture 1.4) 63x oil objective. Z sections were taken every 1 mm Images were analyzed using ImageJ software.

Fly Embryo and S2 collection and immunostaining

Drosophila melanogaster flies were raised in standard corn meal yeast extract medium at 25°C. The Oregon-R line was obtained from A. Bardin's laboratory.

For Western blot experiments, 0-12h old Drosophila embryos grown at 25°C were dechorionated and taken up in ice-cold PBS buffer containing 0.01% Triton. Embryos were homogenized with a homogenizer in 2x SDS–Laemmli buffer. For Western blot experiments on S2 cells, cells were detached from a flask, wash once in PBS, resuspended in lysis buffer (10 mM Tris pH8, 150 mM NaCl, 50 mM KCl, 0,3% Triton, 1mM EDTA, 1mM DTT and added 5x SDS Laemmli buffer.

Immunostaining of polytene chromosome

Polytene chromosome-staining procedures were adapted from a previously described protocol (Lavrov et al., 2004). For Jarid2-K116me2/PH double immunostaining, rabbit anti-Jarid-K116me2 and goat anti-PH antibodies were used.

RT-qPCR

Total RNA was isolated using the Rneasy Mini Kit (Qiagen). cDNA was synthetized using High Capacity cDNA RT kit (4368814-Applied Biosystems) and quantitative PCR was performed with technical triplicate using SYBR green reagent (Roche) on a ViiA7 equipment (Applied Biosystems). At least three biological independent experiments were performed for each assay and negative controls RT are always included. Primers sequences are provided below.

ChIP

ChIPs were performed as described previously (Margueron et al., 2008). Cell confluence and amount of starting material were kept constant by plating defined number of cells the day before cross-linking. Primers sequences for ChIP and RT-qPCR are provided below:

Name	Application	Sequence	
**Luc_FW**	ChIP	GTGTTGGGCGCGTTATTTAT	
**Luc_Rv**	ChIP	TACGGTAGGCTGCGAAATGT	
**Oct4_FW**	RT-qPCR	CTCCCGAGGAGTCCCAGGACAT	*Shen et al, 2009*
**Oct4_Rv**	RT-qPCR	GATGGTGGTCTGGCTGAACACCT	*Shen at al, 2009*
**Gata4_FW**	RT-qPCR	CACAAGATGAACGGCATCAACC	*Pasini et al, 2010*
**Gata4_Rv**	RT-qPCR	CAGCGTGGTGGTGGTAGTCTG	*Pasini et al, 2010*
**Pax3_FW**	RT-qPCR	TCCCATGGTTGCGTCTCTAAG	*Pasini et al, 2010*
**Pax3_Rv**	RT-qPCR	CTCCACGTCAGGCGTTGTC	*Pasini et al, 2010*
**Foxa2_Fw**	RT-qPCR	GATGGAAGGGCACGAGCC	*Pasini et al, 2010*
**Foxa2_Rv**	RT-qPCR	GTATGTGTTCATGCCATTCATCCC	*Pasini et al, 2010*
**Nestin_Fw**	RT-qPCR	GCCTATAGTTCAACGCCCCC	*Pasini et al, 2010*
**Nestin_Rv**	RT-qPCR	AGACAGGCAGGGCTAGCAAG	*Pasini et al, 2010*
**Olig1_Fw**	RT-qPCR	TGAATCCCACCTGTTTAGAGCC	*Pasini et al, 2010*
**Olig1_Rv**	RT-qPCR	CGATGCTCACGGATACGAGAATAG	*Pasini et al, 2010*

ChIP-Seq

ChIP was performed as described above starting from 25 μg of chromatin; magnetic Dynabeads coupled to Protein A were used for the IP (Invitrogen). Sonication was performed to obtain fragment size of 150-300 bp. Libraries were prepared accordingly to manufactures (TruSeq ChIP sample Prep Kit, Illumina). Sequencing was performed on a Illumina Hi-Seq 2500. Single-end 100bp reads were mapped on the Mouse reference genome (mm9) using the Bowtie2 software (Langmead and Salzberg, 2012) allowing one mismatch in the seed (22bp) and reporting one location in case of multiple mapping hits. PCR duplicates were then removed using PicardTools (v1.65, http://picard.sourceforge.net).

Jarid2 and Jarid2-K116me2 ChIP-seq:

Peak calling was performed with MACS (v1.4.2, (Zhang et al., 2008)) with default parameters, using the Input sample as control. Significant peaks were identified with a minimum FDR of 0,15%. The HOMER software (v3.17, http://biowhat.ucsd.edu/homer/) was used to analyze peaks results. Overlapping peaks between the two conditions were detected, quantified and annotated. A gene Ontology analysis was performed with genes near to peaks and the read count histograms around TSS (+/- 3kb) were generated. The genome tracks were generated using the HOMER software by a normalizing library size of 40M reads.

H3K27me3 ChIP-seq:

Peak calling was performed with MACS2 (v2.0.10, (Zhang et al., 2008)) with a DNA fragment size estimated during library preparation, using the Input sample as control. The *broad* parameter was used to call for modified-histone enriched regions. Significant peaks were identified with a minimum FDR of 5%. The Bioconductor Diffbind package (v1.6.2, (Ross-Innes et al., 2012)) was used to detect differential binding sites, with a minimum FDR of 5% and a minimum fold change of 1.5. The set of peaks used to compare undifferentiated ES and EB cell lines was defined using the higher quality samples (replicates 2).

Peaks called differential between ES and EB in one specific condition were annotated with HOMER (v4.3). Genome ontology analysis was done with all differential peaks. Genome tracks were generated using the HOMER software with a DNA fragment size estimated during library preparation.

4) Supplemental Bibliography

Langmead, B., and Salzberg, S.L. (2012). Fast gapped-read alignment with Bowtie 2. Nature methods *9*, 357-359.

Li, G., Margueron, R., Ku, M., Chambon, P., Bernstein, B.E., and Reinberg, D. (2010). Jarid2 and PRC2, partners in regulating gene expression. Genes & development *24*, 368-380.

Margueron, R., Justin, N., Ohno, K., Sharpe, M.L., Son, J., Drury, W.J., 3rd, Voigt, P., Martin, S.R., Taylor, W.R., De Marco, V., *et al.* (2009). Role of the polycomb protein EED in the propagation of repressive histone marks. Nature *461*, 762-767.

Margueron, R., Li, G., Sarma, K., Blais, A., Zavadil, J., Woodcock, C.L., Dynlacht, B.D., and Reinberg, D. (2008). Ezh1 and Ezh2 maintain repressive chromatin through different mechanisms. Molecular cell *32*, 503-518.

Pasini, D., Cloos, P.A., Walfridsson, J., Olsson, L., Bukowski, J.P., Johansen, J.V., Bak, M., Tommerup, N., Rappsilber, J., and Helin, K. (2010). JARID2 regulates binding of the Polycomb repressive complex 2 to target genes in ES cells. Nature *464*, 306-310.

Poullet, P., Carpentier, S., and Barillot, E. (2007). myProMS, a web server for management and validation of mass spectrometry-based proteomic data. Proteomics *7*, 2553-2556.

Ross-Innes, C.S., Stark, R., Teschendorff, A.E., Holmes, K.A., Ali, H.R., Dunning, M.J., Brown, G.D., Gojis, O., Ellis, I.O., Green, A.R., *et al.* (2012). Differential oestrogen receptor binding is associated with clinical outcome in breast cancer. Nature *481*, 389-393.

Zhang, Y., Liu, T., Meyer, C.A., Eeckhoute, J., Johnson, D.S., Bernstein, B.E., Nusbaum, C., Myers, R.M., Brown, M., Li, W., *et al.* (2008). Model-based analysis of ChIP-Seq (MACS). Genome biology *9*, R137.
